# Supplementary material for: Can clinical audits be enhanced by pathway simulation and machine learning? An example from the acute stroke pathway
Source: BMJ Open. 2019 Sep 17;9(9):e028296. doi: 10.1136/bmjopen-2018-028296 (PMC6756466; doi:10.1136/bmjopen-2018-028296)
Supplement: Supplementary data [file bmjopen-2018-028296supp001.pdf]

Can clinical audit be enhanced by pathway simulation and machine learning? An example from the acute stroke pathway.

Supplement

1 GitHub code repository

Both the pathway simulation and the machine learning models use Free Open Source Software (both models are based on Python). All code for the model (with anonymous patient data) is available at https://github.com/MichaelAllen1966/stroke_thrombolysis_pathway

2 Data

Anonymous SSNAP data was obtained from 7 hospitals in England, for patients with a confirmed stroke over a period of two years (2013-2014) for each hospital. These data were secondary data, collected during routine care. No patient identifiable information was obtained.

For the pathway simulation model, the dataset contained 7,871 patient records with complete data for 12 parameters regarding their characteristics and time stamped pathway location. These data represent out-of-hospital onset of stroke (which account for 94% of all admissions recorded in the SSNAP data used).

For machine learning, only those patients with a completed NIH Stroke Scale and who had at least 30 minutes left to give thrombolysis were used (1,862 patients). As a precaution to maintain complete patient anonymity 17 patients aged under 40 had their age censored to 40, and 6 patients over the age of 100 had their age censored to 100.

2.1 Patient characteristics

Table 1 shows the characteristic of patients used for modelling. The clinical pathway simulation model used all patients with out-of-hospital onset, whereas as the machine learning thrombolysis decision model was based on those patients scanned with 30 minutes left for treatment, and who had a NIHSS score.

Allen M, *et al. BMJ Open* 2019; 9:e028296. doi: 10.1136/bmjopen-2018-028296

Table 1: Characteristics of patients used in the models

	Mean (+standard deviation where appropriate)	
	All out of hospital admissions	All patients with 30 minutes left to treat after scan (and NIHSS recorded)
Patients	7871	1862
Thrombolysis given	0.09	0.40
Male	0.48	0.52
Age	76 (13)	74 (13)
Age 80+	0.48	0.37
Onset time known	0.55	1.00
Arrive by ambulance	0.85	0.95
Arrival within 4 hours of known onset	0.38	1.00
Age 80+ 2.5 hour arrival, others 4hr arrival	0.35	1.00
Scanned	0.99	1.00
# Comorbidities	1.00	1.18
2+ comorbidities	0.36	0.35
Congestive HF	0.05	0.05
Hypertension	0.48	0.47
Atrial Fib	0.21	0.22
Diabetes	0.17	0.16
Previous TIA	0.30	0.28
Co-mordity	0.72	0.71
Antiplatelet	0.38	0.08
Anticoag before stroke	0.50	0.10
NIHSS recorded	0.66	1.00
NIHSS on Arrival	8.0 (7.7)	10.4 (7.8)
Onset to scan with 30 min left	0.25	1.00
Arrival to stroke unit (min)	858 (2386)	86 (38)
Arrival to scan	375 (1389)	29 (27)
Onset to scan	969 (11119)	116 (44)
Scan to thrombolysis	42 (27)	42 (27)
Arrival to thrombolysis	69 (35)	68 (34)
Onset to thrombolysis	156 (69)	148 (47)

Allen M, *et al. BMJ Open* 2019; 9:e028296. doi: 10.1136/bmjopen-2018-028296

Table 2 shows key patients characteristics, summarised by hospital, for patients with 30 minutes left to treat after scan (and NIHSS recorded).

Table 2: Key patients characteristics, summarised by hospital, for patients with 30 minutes left to treat after scan (and NIHSS recorded)

	Hospital						
	1	2	3	4	5	6	7
Age	74 (13)	75 (14)	72 (12)	75 (12)	75 (11)	74 (13)	75 (12)
Age 80+	0.38	0.42	0.25	0.41	0.38	0.34	0.39
Male	0.51	0.49	0.54	0.49	0.43	0.54	0.56
NIHSS on Arrival	11.8 (8.0)	9.5 (8.1)	10.6 (6.9)	11.2 (8.3)	12.2 (7.9)	9.7 (6.8)	9.0 (7.4)

3 Pathway simulation model

3.1 High level model description

The simulation model mimics the flow of individual patients through the acute stroke pathway as shown in figure 1). The simulation model builds a 'virtual population' of patients which travel through the model. The characteristics of the patients are taken from sampling from distributions based on the patient-level SSNAP data for each individual hospital.

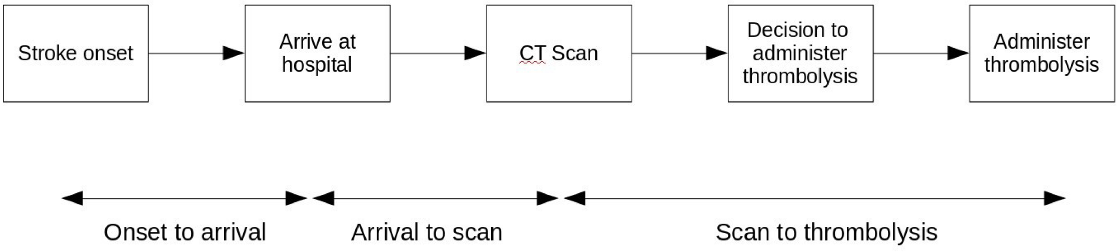

Figure 1: High level view of patient flow through model

For a patient to receive thrombolysis in the model the patient must:

1. Arrive within four hours of known onset
2. Receive a scan within 4 hours of arrival and receive a scan with at least 20 minutes left to treat with thrombolysis (onset-to-treatment time limits are 270 minutes for patients aged less than 80, and 180 minutes for patients aged 80+).
3. Have an ischaemic stroke and be considered eligible for thrombolysis

Allen M, *et al. BMJ Open* 2019; 9:e028296. doi: 10.1136/bmjopen-2018-028296

For patients receiving thrombolysis the probability of an additional good outcome is calculated according to the analysis of Emberson *et al.[1]*.

The model runs in Python (using NumPy arrays to represent a population of patients). Each run of the model mimics 100 independent one year of admissions to a given hospital.

3.2 Model outputs

For each scenario, the model reports for the following (from 100 independent years):

- Mean, standard deviation, 5th percentile, lower quartile, median, upper quartile, and 95th percentile of:
  - Baseline good outcomes (MRS0-1)
  - Thrombolysis use (% of all admissions)
  - Additional good outcomes per 1,000 admissions

3.3 Hospital performance parameters used in the pathway simulation model

The parameters used in the thrombolysis pathway simulation model are shown in table 3. All parameters are derived from data recorded in SSNAP.

Table 3: Parameters used in the stroke pathway simulation model (patients with in-hospital stroke onset excluded)

Parameter	Distribution	Range used (base case)
Allowed onset-to-treatment time for age up to 80 (mins)	Fixed	270
Allowed onset-to-treatment time for age 80 +(mins)	Fixed	180
Age 80+ (patients arriving within 4 hours of known onset)	Bernoulli	0.363-0.523
Arrivals per year	fixed	300-800
Onset time known	Bernoulli	0.432-0.738
Onset-to-arrival <4hrs	Bernoulli	0.677-0.706
Onset-to-arrival time (mins)	Log (ln) normal	Mean (ln): 4.417-4.609<br>StdDev (ln): 0.480-0.573
Arrival-to-scan <4hrs	Bernoulli	0.783-0.979
Arrival-to-scan time (mins)	Log (ln) normal	Mean (ln): 2.477-3.789<br>StdDev (ln): 0.748-1.271
Ischaemic stroke	Bernoulli	0.770-0.896
Eligible for thrombolysis	Bernoulli	0.342-0.577
Scan-to-thrombolysis (mins)	Log (ln) normal	Mean (ln): 23.087-3.80<br>StdDev (ln): 0.560-0.863

Allen M, *et al. BMJ Open* 2019; 9:e028296. doi: 10.1136/bmjopen-2018-028296

Notes:

1. *The allowed onset-to-treatment time for patients aged up to 80 is 4.5 hours.*
2. *The allowed onset-to-treatment time for patients aged 80+ is 3 hours.*
3. *The proportion of patients aged 80+ varies between hospitals.*
4. *The number of arrivals is the number of confirmed stroke patients in SSNAP per year*
5. *'Onset time known' is binary yes/no. Known onset time may be recorded as precise or estimated in SSNAP. Those without a known onset time cannot be treated with thrombolysis in the model.*
6. *'Onset to arrival <4hrs' is the proportion of patients with known stroke onset that arrive within 4 hours of onset. Those arriving more than 4 hours after onset cannot be treated with thrombolysis in the model.*
7. *'Onset to arrival time' is a log-normal distribution. It is applied only to those patients arriving within 4 hours of known stroke onset.*
8. *'Arrival to scan time <4hrs' is the proportion of patients (those with known stroke onset time and arriving within 4 hours of onset) that receive a CT head scan within 4 hours of arrival.*
9. *'Arrival to scan time' is a log-normal distribution. It is applied only to those patients arriving within 4 hours of known stroke onset, and receiving a scan within 4 hours of arrival.*
10. *'Ischaemic stroke' is the proportion of patients with ischaemic (rather than haemorrhagic stroke). It is applied only to those patients arriving within 4 hours of known stroke onset, and receiving a scan within 4 hours of arrival.*
11. *'Eligible for thrombolysis' is the proportion of ischaemic stroke patients (arriving within 4 hours of known stroke onset, and scanned within 4 hours of arrival) who are considered clinically eligible for thrombolysis. This figure is obtained by examining the proportion of ischaemic stroke patients who are scanned with at least 30 minutes left to receive thrombolysis who are given thrombolysis.*
12. *'Scan to thrombolysis' is a log-normal distribution. It is applied only to those patients arriving within 4 hours of known stroke onset, receiving a scan within 4 hours of arrival, and are ischaemic strokes considered eligible for thrombolysis*

The value of parameters used for each hospital's base case is shown in table 4.

Allen M, *et al. BMJ Open* 2019; 9:e028296. doi: 10.1136/bmjopen-2018-028296

Table 4: Model input parameters for each of the seven hospitals in the study

Hospital	1	2	3	4	5	6	7
arrivals per year	600	600	800	700	300	350	700
Age 80+ (for patients arriving within 4hrs of known onset)	0.492	0.523	0.363	0.486	0.422	0.490	0.475
onset known	0.679	0.537	0.541	0.432	0.460	0.738	0.562
known arrival within 4hrs	0.684	0.698	0.695	0.677	0.697	0.706	0.704
onset arrival mins μ	4.568	4.471	4.543	4.609	4.417	4.539	4.445
onset arrival mins σ	0.513	0.539	0.480	0.519	0.550	0.505	0.573
scan within 4 hrs	0.844	0.783	0.940	0.979	0.848	0.845	0.910
arrival scan arrival mins μ	3.645	3.421	3.789	2.477	3.660	3.409	3.151
arrival scan arrival mins σ	0.905	1.097	0.748	1.271	0.812	0.980	1.012
ischaemic stroke	0.865	0.896	0.820	0.864	0.831	0.770	0.859
ischaemic eligible	0.557	0.386	0.383	0.344	0.469	0.511	0.342
scan needle mins μ	3.087	3.547	3.800	3.695	3.522	3.461	3.686
scan needle mins σ	0.863	0.732	0.583	0.560	0.608	0.732	0.587
thrombolysis rate (actual)	0.137	0.080	0.084	0.070	0.085	0.145	0.092

3.3.1 Correlation between input parameters (patient level)

Table 5 shows correlation between model input parameters. Of the key steps in the model (arrival to scan time, scan to thrombolysis, proportion of patients receiving thrombolysis if there is time to treat), the strongest correlation (R-square) is -0.16.

Allen M, *et al. BMJ Open* 2019; 9:e028296. doi: 10.1136/bmjopen-2018-028296

Table 5: Correlation between model input parameters (for patients arriving at an acute stroke unit within 4 hours of stroke onset)

	A	B	C	D	E	F	G	H
A	NA	0.03	-0.04	0.04	-0.30	-0.02	-0.12	-0.04
B	0.03	NA	-0.13	0.11	-0.49	-0.04	-0.24	-0.13
C	-0.04	-0.13	NA	-0.65	0.51	0.00	0.21	NA
D	0.04	0.11	-0.65	NA	-0.36	0.01	-0.15	-0.16
E	-0.30	-0.49	0.51	-0.36	NA	0.06	0.35	NA
F	-0.02	-0.04	0.00	0.01	0.06	NA	NA	NA
G	-0.12	-0.24	0.21	-0.15	0.35	NA	NA	NA
H	-0.04	-0.13	NA	-0.16	NA	NA	NA	NA

A: Age 80+
B: Onset to arrival (min)
C: Scanned within 4hrs arrival
D: Arrival to scan
E: Onset to scan with 30 min left
F: Scan to thrombolysis
G: Thrombolysis given
H: Thrombolysis given if 30 minutes left to treat

3.4 Model steps

The model loads input parameters for multiple scenarios. For each scenario the following steps are performed:

1. Create an array representing all patient arrivals for one year (note: in the following steps all values are assigned to all patients, though they are only applied as appropriate)
2. Assign patient as aged 80+ or not (distribution os for patients who arrive within four hours of known onset)
3. Assign allowed onset-to-needle (depending on age group)
4. Assign onset time known or not
5. Assign onset to-arrival within 4 hours of known stroke onset
6. Assign onset-to-arrival time
7. Assign arrival to scan within 4 hours or not
8. Assign arrival to scan times
9. Assign time left for thrombolysis decision
10. Assign whether patients has all of: onset time known, onset-to-arrival <4hrs, arrival to scan <4hrs, and time left to receive thrombolysis (20 minutes time window remaining)

Allen M, *et al. BMJ Open* 2019; 9:e028296. doi: 10.1136/bmjopen-2018-028296

11. Assign whether patient has ischaemic stroke or not
12. Assign whether patient is eligible for thrombolysis
13. Assign desire to treat (based on steps 10, 12 & 12 all being positive)
14. Assign scan to needle time
15. Check onset-to-needle time is within licence time (+15 minutes to allow for over-run in the decision-to-treat to treatment stage).
16. Assign thrombolysis given (based on steps 13 & 15 both being positive)
17. Set basline probability of good outcome (based on age group), and convert to odds.
18. Assign odds-ratio of good outcome based on giving thrombolysis at assigned onset-to-treatment tie
19. Adjust odds of good outcome based on assigned odds-ratio in step 18, Convert back to probability.
20. Calculate additional probability of good outcome if given thrombolysis
21. Sum additional probabilities of good outcomes for all those treated (step 16)
22. Save proportion of admissions given thrombolysis (step 16) and the number of additional good outcomes (step 21).

3.5 The relationship between time to treatment and outcome

The effectiveness of thrombolysis as a function of time to treatment is taken from Emberson et al. [1], and is shown in figure 2. The model uses odds-ratio of a good outcome (Modified Rankin Scale 0-1). The baseline probabilities of a good outcome for patients aged less than 80, and 80+ are 0.350 and 0.132 respectively (equivalent odds = 0.538 and 0.152). which are taken from the same paper.

We apply an allowable onset-to-treatment time of 270 minutes for patients aged less than 80, and 180 minutes for patients aged 80+.

Allen M, *et al. BMJ Open* 2019; 9:e028296. doi: 10.1136/bmjopen-2018-028296

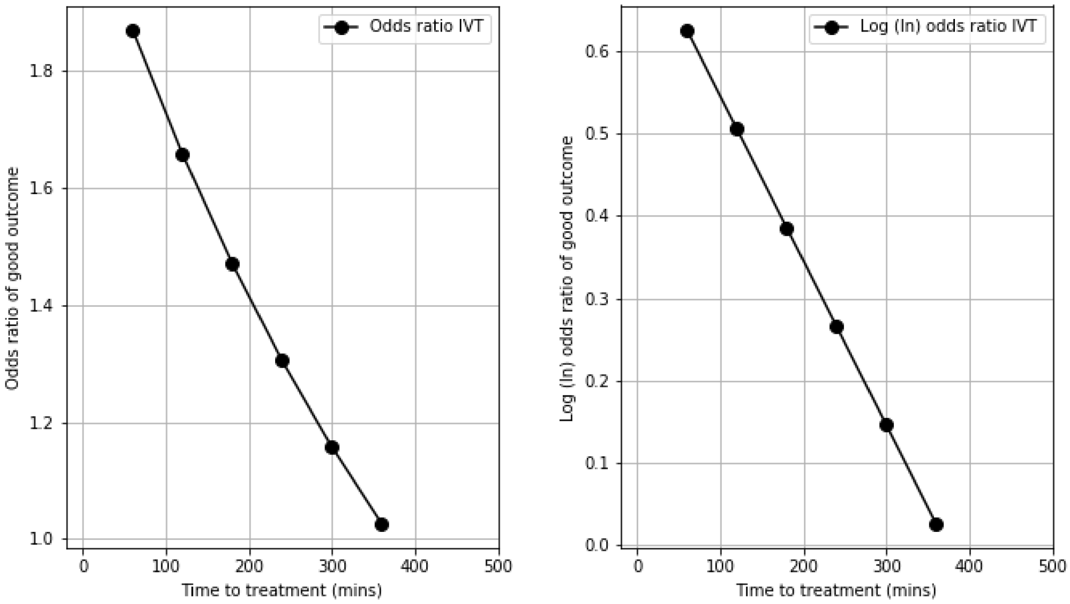

Figure 2: The decay of effectiveness of IVT as a function of time to treatment, shown as decay in odds (left) or log odds (right).

3.6 Model validation

The pathway simulation model was validated by 1) random bootstrap sampling varying overall thrombolysis use, comparing actual to predicted thrombolysis use, and 2) comparing actual and predicted thrombolysis use and speed across the seven hospitals.

In the random sampling (figure 3), 600 patients (typical of an acute stroke unit) were sampled randomly from either the thrombolysis group or the no-thrombolysis group, to give varying samples with an overall thrombolysis use rate of 1-25%. 100 model runs were performed. The model showed very good correlation (R-square 0.99) between actual and predicted values though the model slightly under-predicted actual thrombolysis use with predicted thrombolysis being, on average, 89% that of actual thrombolysis use.

Allen M, *et al. BMJ Open* 2019; 9:e028296. doi: 10.1136/bmjopen-2018-028296

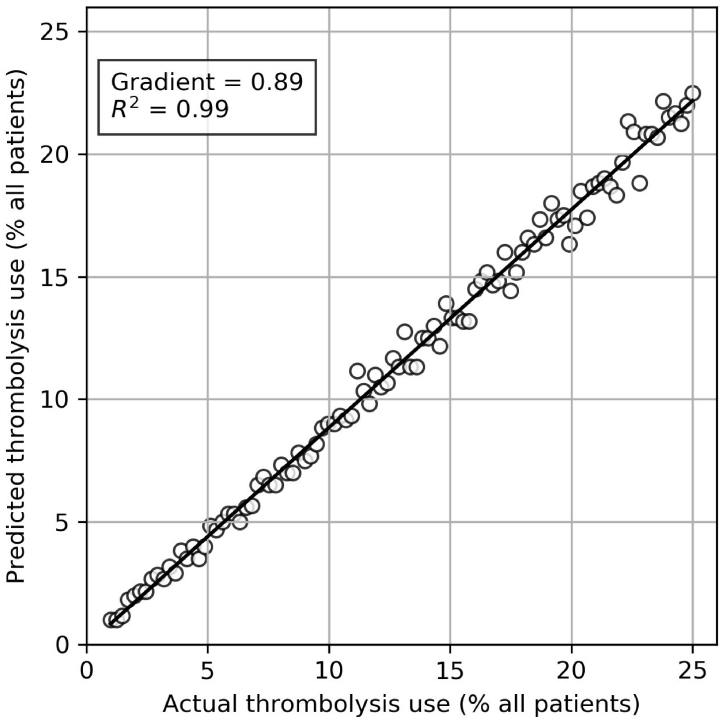

Figure 3: Validation of the pathway simulation model, comparing actual to modelled (predicted) thrombolysis based on random sampling of all data. Samples were 600 points (representing typical sacute troke unit admission numbers) chosen randomly with resampling from patients given or not given thrombolysis to create a range of thrombolysis use examples. Points show mean predicted thrombolysis use for all confirmed stroke patients arriving at hospital, with bars showing 5th and 95th percentiles from 100 runs, with each run modelling one year.

The model was further validated by comparing modelled (predicted) use of thrombolysis with actual use of thrombolysis. Actual use of thrombolysis was based on modelling a one year period, with replicates of 100 runs (each with different random number seeds) in order to determine expected year-to-year variation. The model showed very good correlation (R-square 0.96) between actual and predicted values though the model again slightly under-predicted actual thrombolysis use with predicted thrombolysis being, on average, 90% that of actual thrombolysis use (table 6).

Allen M, *et al. BMJ Open* 2019; 9:e028296. doi: 10.1136/bmjopen-2018-028296

Table 6: Comparison of actual vs. modelled hospital performance

**Hospital**	**1**	**2**	**3**	**4**	**5**	**6**	**7**
Actual thrombolysis (%)	13.7	8.0	8.4	7.0	8.5	14.5	9.2
Model thrombolysis (%)	12.9	7.1	7.6	6.3	8.1	12.0	8.0
Actual onset to thrombolysis (hrs)	2.4	2.6	2.9	2.5	2.3	2.5	2.5
Model onset to thrombolysis (hrs)	2.6	2.6	3.0	2.6	2.7	2.6	2.5

The difference between predicted and actual thrombolysis use is largely explained by the observation that 8% of thrombolysis in the SSNAP data set was given outside of the assumed allowable times for thrombolysis in the model (4.5 hrs for patients aged <80, and 3 hrs for patients aged 80+); the model applies a stricter time cut-off than clinicians allow in reality.

3.7 Alternative model scenarios

Though many different ‘what if?’ scenarios may be tested in the model we have reported on key alternative scenarios?

1. Door-to-needle time 30 minutes for 90% patients (the other 10% do not receive a scan within time to treat with thrombolysis). Door-to-needle time is split equally between arrival-to-scan and scan-to-thrombolysis?
2. Of those with ischaemic stroke, and who receive a scan with time to treat, 60% are treated with thrombolysis (from the analysis by Bembenek *et al*. [2]).
3. Stroke onset time is determined for 77% of patients (upper quartile point in SSNAP 2016/17).
4. Combinations of the above

Additionally, in place of scenario 2, thrombolysis rate may be determined according to the method based on machine learning (see below), to allow for stroke patient population differences between hospitals.

4 Machine learning

All machine learning methods and validation was coded in Python using the SciKit Learn machine learning [3].

All models are *supervised learning* models, and predict whether an individual patient would receive thrombolysis or not given a set of training data with features and known label (whether thrombolysis was given). The machine learning models evaluated are:

- Logistic regression with regularisation [4].

Allen M, *et al. BMJ Open* 2019; 9:e028296. doi: 10.1136/bmjopen-2018-028296

- Random Forests [5].
- Support Vector Machines: either a linear model was used, or a model using feature transformation by a radial basis function (rbf) kernel [6].
- A Feed-Forward Neural Network (results report a network based on two hidden layers of 50 and 5 nodes, after optimisation of the network size) [7].

Meta-parameters for each model may be found in the GitHub code repository, in the file machine_learning_all_techniques.py

4.1 Model features

Model features were set for each patient (anonymous data only was used) from SSNAP (Sentinel Stroke National Audit Programme).

The inclusion criteria for patients was the patient had been scanned with 30 minutes left to give thrombolysis (allowing 4.5 hours and 3 hours from onset to treatment for patients aged under 80, and 80+, respectively). Details of the individual fields are shown in table 7.

Allen M, *et al. BMJ Open* 2019; 9:e028296. doi: 10.1136/bmjopen-2018-028296

Table 7: SSNAP fields used in machine learning model

Feature	Type	Feature	Type
Hosp_1	Binary	Anticoag before stroke_1	Binary
Hosp_2	Binary	Anticoag before stroke_NK	Binary
Hosp_3	Binary	Stroke severity group_1. No stroke symptoms	Binary
Hosp_4	Binary	Stroke severity group_2. Minor	Binary
Hosp_5	Binary	Stroke severity group_3. Moderate	Binary
Hosp_6	Binary	Stroke severity group_4. Moderate to severe	Binary
Hosp_7	Binary	Stroke severity group_5. Severe	Binary
Male	Binary	Stroke Type_I	Binary
Age (years)	Integer	Stroke Type_PIH	Binary
80	Binary	S2RankinBeforeStroke	Integer
Onset Time Known Type_BE	Binary	S2NihssArrival	Integer
Onset Time Known Type_NK	Binary	S2NihssArrivalLocQuestions	Integer
Onset Time Known Type_P	Binary	S2NihssArrivalLocCommands	Integer
# Comorbidities	Integer	S2NihssArrivalBestGaze	Integer
2+ comorbidotes	Binary	S2NihssArrivalVisual	Integer
Congestive HF	Binary	S2NihssArrivalFacialPalsy	Integer
Hypertension	Binary	S2NihssArrivalMotorArmLeft	Integer
Atrial Fib	Binary	S2NihssArrivalMotorArmRight	Integer
Diabetes	Binary	S2NihssArrivalMotorLegLeft	Integer
TIA	Binary	S2NihssArrivalMotorLegRight	Integer
Co-mordity	Binary	S2NihssArrivalLimbAtaxia	Integer
Antiplatelet_0	Binary	S2NihssArrivalSensory	Integer
Antiplatelet_1	Binary	S2NihssArrivalBestLanguage	Integer
Antiplatelet_NK	Binary	S2NihssArrivalDysarthria	Integer
Anticoag before stroke_0	Binary	S2NihssArrivalExtinctionInattention	Integer

4.2 Machine learning validation

The machine learning model was validated using stratified ten-fold validation [8], where the data is split into 10 subsets, and the model run 10 times (with each model run using 9 subsets for training and 1 subset held back for testing, with all data present in a test subset once and only once). For validation all hospitals were combined, with the hospital as an input parameter in the model.

Table 8 shows performance of different machine learning models (Support Vector Machines models were preformed with linear or radial basis function kernels). All models used the SciKit Learn library for Python. The Random Forest model was chosen for the work described in the paper.

Allen M, *et al. BMJ Open* 2019; 9:e028296. doi: 10.1136/bmjopen-2018-028296

The models were evaluated for:

- *Accuracy*: The proportion of thrombolysis decisions correctly predicted.
- *Sensitivity* (also called *recall* or *true positive rate)*: The proportion of positive thrombolysis decisions correctly predicted.
- *Specificity*: The proportion of negative thrombolysis decisions correctly predicted. The false *positive rate* is equal to 1-specificity.
- ROC area: The area of the operator-receiver characteristic curve, a plot of *true positive rate* against *false positive rate* [9].

Table 8: Performance of machine learning models (Mean and standard deviation for 10 k-fold training/test splits). Random Forest was chosen for its highest overall accuracy and ROC.

	accuracy	sensitivity	specificity	ROC area
Logistic regression	0.807 (0.010)	0.788 (0.015)	0.819 (0.012)	0.882 (0.005)
**Random Forest**	**0.819 (0.010)**	**0.792 (0.017)**	**0.837 (0.011)**	**0.889 (0.007)**
SVM (linear)	0.807 (0.008)	0.820 (0.012)	0.798 (0.012)	0.880 (0.005)
SVM (rbf)	0.813 (0.006)	0.820 (0.013)	0.808 (0.008)	0.880 (0.006)
Neural Network	0.807 (0.010)	0.794 (0.017)	0.816 (0.012)	0.882 (0.005)

Allen M, *et al. BMJ Open* 2019; 9:e028296. doi: 10.1136/bmjopen-2018-028296

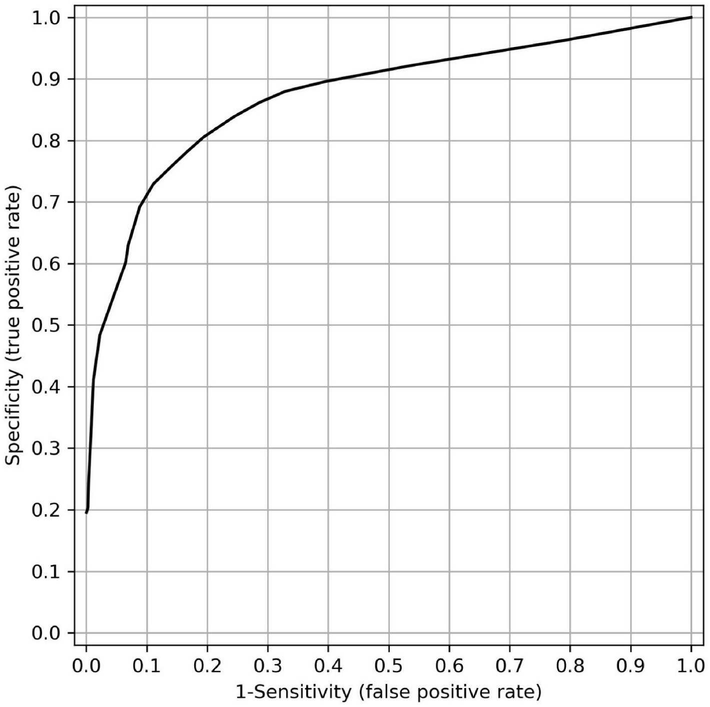

Figure 4: Receiver Operator Curve (ROC) for the Random Forest model

Figure 4 shows the ROC for Random Forest model, which plots true positive rate (sensitivity, or recall) against false positive rate (1-specificity).

4.3 Learning curve for Random Forest

Figure 5 shows the learning curve for the Random Forest model. The learning rate shows how accuracy increases with the size of the training data set, and helps to establish whether more data is likely to lead to increased accuracy of the model. The accuracy of Random Forest model (assess by stratified 10-fold validation, with the training set reduced to the required size) increases rapidly to 79% accuracy with 400 data points, and then there is a shallow incline of improvement of accuracy up to our maximum available training data set size of 1600 data points. A larger data set may lead to further small increase in accuracy.

Allen M, *et al. BMJ Open* 2019; 9:e028296. doi: 10.1136/bmjopen-2018-028296

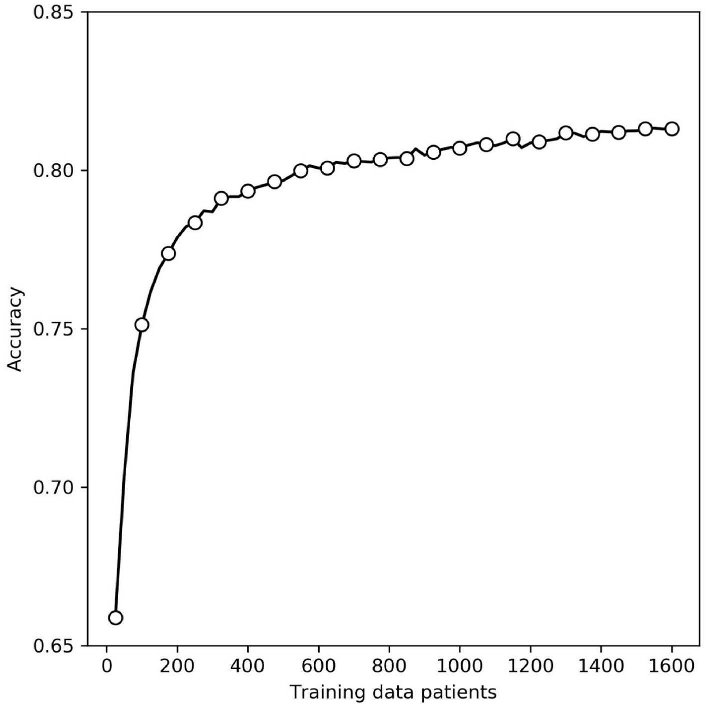

Figure 5: Learning curve for the Random Forest model. Accuracy was measured using stratified 10-fold validation.

4.4 Machine learning alternative scenarios

A machine learning model may be trained on a subset of patients to investigate how the difference in thrombolysis use between hospitals may be proportionally attributed to either the hospital or the local patient population. Table 9 shows the predicted use of thrombolysis in a set of patients that attend one hospital, based on decisions made from training at another hospital.

Taking hospital 7 as an example, the current thrombolysis use rate is 31% (for patients with 30 minutes left to receive thrombolysis). But of the Random Forest model is trained on decisions made at hospital 1, then 45% of the patients attending hospital 7 are predicted to receive thrombolysis. This is still lower than the 52% thrombolysis use rate in hospital 7, suggesting that the difference in thrombolysis rates between hospital 7 and 1 are due to differences in both decision-making (with hospital 1 being more inclined to use thrombolysis) and patients (with patients attending hospital 7 being less suitable for thrombolysis).

Alternatively the table shows the likely thrombolysis use rate at hospital 7 given alternative patient populations. If hospital 7 received patients currently attending hospital 1 then the model predicts thrombolysis use would be 40%. This is higher than the 31% current use of thrombolysis, but lower than the 52% current performance at hospital 1, again demonstrating that the use of thrombolysis depends on both the hospital and the patient cohort, and that not all hospitals should be expected to

achieve the same thrombolysis use rate (even when the patient cohort is restricted to those with time to thrombolyse). One possible explanation for these results is that the average stroke severity for hospital 7 is lower than than that for hospital 1 (9.0 vs 11.8, table 2), and that all hospitals may be less inclined to treat more mild strokes with thrombolysis, but that hospital 1 is more inclined than hospital 7 to treat more mild strokes with thrombolysis.

Table 9: Predicted thrombolysis use (for patients with patients scanned with time left to thrombolysis) if the decision to give thrombolysis is based on decisions made by a Random Forest model trained at different hospitals. The columns therefore represent the likely difference in thrombolysis use due to differences in decision making. Hospital 7 is highlighted as an example described in the text.

		**Actual thrombolysis use by hospital**						
		**1**	**2**	**3**	**4**	**5**	**6**	**7**
		52	35	48	33	49	44	31
		**Hospital patients actually attended**						
		1	2	3	4	5	6	**7**
**Hospital used to train model**	**1**	52	42	58	50	67	57	45
	**2**	48	35	55	36	46	37	29
	**3**	53	38	48	46	58	41	34
	**4**	40	28	48	33	52	29	26
	**5**	50	36	50	40	49	45	37
	**6**	49	32	55	44	59	44	39
	**7**	42	23	42	31	50	36	31

It should be noted that table 9 represents predictions of *counter-factual scenarios* that can never be validated.

5 References

1 Emberson J, Lees KR, Lyden P, *et al.* Effect of treatment delay, age, and stroke severity on the effects of intravenous thrombolysis with alteplase for acute ischaemic stroke: A meta-analysis of individual patient data from randomised trials. *The Lancet* 2014;**384**:1929–1935. doi:10.1016/S0140-6736(14)60584-5

2 Bembenek J, Kobayashi A, Sandercock P, *et al.* How many patients might receive thrombolytic therapy in the light of the ECASS-3 and IST-3 data? *Int J Stroke* 2010;**5**:430–1. doi:10.1111/j.1747-4949.2010.00479.x

3 Pedregosa F, Varoquaux G, Gramfort A, *et al.* Scikit-learn: Machine Learning in Python. *J Mach Learn Res* 2011;**12**:2825–30.

4 Yu H-F, Huang F-L, Lin C-J. Dual coordinate descent methods for logistic regression and maximum entropy models. *Mach Learn* 2011;**85**:41–75. doi:10.1007/s10994-010-5221-8

Allen M, *et al. BMJ Open* 2019; 9:e028296. doi: 10.1136/bmjopen-2018-028296

5 Breiman, L. Random Forests. *Mach Learn*;**45**:5–32.

6 Fan R-E, Chang K-W, Hsieh C-J, *et al.* LIBLINEAR: A Library for Large Linear Classification. *J Mach Learn* 2008;**9**:1871–4.

7 Rumelhart, Daid E., Hinton, Geoffrey, E, Williams, Ronald J. Learning internal representations by error-propagation. In: *Parallel Distributed Processing: Explorations in the Microstructure of Cognition. Volume 1*. MIT Press, Cambridge, MA 1986. 318–62.

8 Stone, M. Cross-validatory Choice and Assessment of Statistical Predictions. *J R Stat Soc Ser B-Methodol* 1974;**36**:111–47.

9 Fawcett T. An introduction to ROC analysis. *Pattern Recognit Lett* 2006;**27**:861–74. doi:10.1016/j.patrec.2005.10.010
