# Supplementary material for: Can clinical audits be enhanced by pathway simulation and machine learning? An example from the acute stroke pathway
Source: BMJ Open. 2019 Sep 17;9(9):e028296. doi: 10.1136/bmjopen-2018-028296 (PMC6756466; doi:10.1136/bmjopen-2018-028296)
Supplement: Author's manuscript [file bmjopen-2018-028296.draft_revisions.pdf]

BMJ Open is committed to open peer review. As part of this commitment we make the peer review history of every article we publish publicly available.

When an article is published we post the peer reviewers' comments and the authors' responses online. We also post the versions of the paper that were used during peer review. These are the versions that the peer review comments apply to.

The versions of the paper that follow are the versions that were submitted during the peer review process. They are not the versions of record or the final published versions. They should not be cited or distributed as the published version of this manuscript.

BMJ Open is an open access journal and the full, final, typeset and author-corrected version of record of the manuscript is available on our site with no access controls, subscription charges or pay-per-view fees (http://bmjopen.bmj.com).

If you have any questions on BMJ Open's open peer review process please email info.bmjopen@bmj.com

BMJ Open

# Can clinical audits be enhanced by pathway simulation and machine learning? An example from the acute stroke pathway.

| | |
|---|---|
| Journal: | *BMJ Open* |
| Manuscript ID | bmjopen-2018-028296 |
| Article Type: | Research |
| Date Submitted by the Author: | 30-Nov-2018 |
| Complete List of Authors: | Allen, Michael; University of Exeter, Medical School<br>Pearn, Kerry; University of Exeter, Medical School<br>Monks, T; University of Southampton<br>Bray, Benjamin D.; Royal Coll Physicians<br>Everson, Richard; University of Exeter, Computer Sciences<br>Salmon, Andrew; University of Exeter<br>James, Martin; Royal Devon & Exeter NHS trust<br>Stein, Ken; University of Exeter Medical School, PenTAG |
| Keywords: | Stroke < NEUROLOGY, thrombolysis, alteplase, simulation, machine learning, health services research |
| | |

SCHOLARONE™
Manuscripts

# Can clinical audits be enhanced by pathway simulation and machine learning? An example from the acute stroke pathway.

Michael Allen*, Honorary Research Fellow, University of Exeter Medical School, St Luke's Campus, Heavitree Road, EXETER, EX1 2LU, UK

Kerry Pearn, Associate Research Fellow, University of Exeter Medical School, St Luke's Campus, Heavitree Road, EXETER, EX1 2LU, UK

Thomas Monks, Principal Research Fellow, NIHR CLAHRC Wessex Data Science Hub, Room AA72 South Academic Block, Southampton General Hospital, SOUTHAMPTON, SO16 6YD

Benjamin Bray, Research Director, Sentinel Stroke National Audit Programme (SSNAP), Kings College, London

Richard Everson, Professor of Machine Learning, Computer Sciences, University of Exeter

Andrew Salmon, Associate Research Fellow, University of Exeter Medical School, St Luke's Campus, Heavitree Road, EXETER, EX1 2LU, UK

Martin James, Consultant Stroke Physician, Royal Devon & Exeter NHS trust, Barrack Road, EXETER, EX2 5DW, UK

Ken Stein, Deputy Director of PenCLAHRC, University of Exeter Medical School, St Luke's Campus, Heavitree Road, EXETER, EX1 2LU, UK

**keywords**: stroke, thrombolysis, alteplase, simulation, machine learning

**artificial intelligence**

*Correspondence to: m.allen@exeter.ac.uk

The Corresponding Author has the right to grant on behalf of all authors and does grant on behalf of all authors, a worldwide licence to the Publishers and its licensees in perpetuity, in all forms, formats and media (whether known now or created in the future), to i) publish, reproduce, distribute, display and store the Contribution, ii) translate the Contribution into other languages, create adaptations, reprints, include within collections and create summaries, extracts and/or, abstracts of the Contribution, iii) create any other derivative work(s) based on the Contribution, iv) to exploit all subsidiary rights in the Contribution, v) the inclusion of electronic links from the Contribution to third party material where-ever it may be located; and, vi) licence any third party to do any or all of the above.

For peer review only

All authors have completed the ICMJE uniform disclosure form at www.icmje.org/coi_disclosure.pdf and declare: no support from any organisation for the submitted work; no financial relationships with any organisations that might have an interest in the submitted work in the previous three years; no other relationships or activities that could appear to have influenced the submitted work.

**Conflicts of interest**: None

**Transparency:** The lead author (Michael Allen) confirms that the manuscript is an honest, accurate, and transparent account of the study being reported; that no important aspects of the study have been omitted.

**Funding**: This study was jointly funded by the National Institute of Health Research (NIHR) Collaboration for Leadership in Applied Health Research and Care for the South West Peninsula, and the South West Academic Health Science Network. The views and opinions expressed in this paper are those of the authors, and not necessarily those of the NHS, the National Institute for Health Research, or the Department of Health.

**Data sharing**: Data and code used in this study is available at https://github.com/MichaelAllen1966/1807_stroke_pathway

**Ethical approval**: This study used anonymous secondary patient data only, collected as part of routine care audit. The study was conducted as part of a local service evaluation and improvement exercise sponsored by the South West Strategic Clinical Network. There was no access to patient identifiable information, no collecting of primary data, and no patient care was altered during the process of this study. There was no patient enrolment into the study. A decision that no formal ethical approval was made by the project steering group with representation from the South West Strategic Clinical Network, the National Institute of Health Research (NIHR) Collaboration for Leadership in Applied Health Research and Care for the South West Peninsula, and the South West Academic Health Science Network.

**Guarantor**: KS

**Contributorship**: MA, KP, MJ and KS conceived the study. RE reviewed and provided guidance on the machine learning aspects. TM and KP reviewed and provided guidance on the simulation aspects. AS conducted literature reviews and provided guidance on methodologies. MA wrote the algorithm code used in this study and performed the primary analysis. MJ and BB informed the base assumptions of the model, provided key literature and reviewed the output. MA produced the first draft of the paper, and all authors contributed significant editing to the paper. KS oversaw all stages of the project.

For peer review only

# Abstract

**Objectives:** To evaluate the application of clinical pathway simulation in machine learning, using clinical audit data, in order to identify key drivers for improving use and speed of thrombolysis at individual hospitals.

**Design:** Computer simulation modelling and machine learning.

**Setting:** Seven acute stroke units.

**Participants:** Anonymised clinical audit data for 7,864 patients.

**Results:** Three factors were pivotal in governing thrombolysis use: (1) the proportion of patients with a known stroke onset time (range 44% to 73%), (2) pathway speed (for patients arriving within 4 hours of onset: per-hospital median arrival-to-scan ranged from 11 to 56 minutes; median scan-to-thrombolysis ranged from 21 to 44 minutes) and (3) predisposition to use thrombolysis (thrombolysis use ranged from 31% to 52% for stroke patients scanned with 30 minutes left to administer thrombolysis). A pathway simulation model could predict the potential benefit of improving individual stages of the clinical pathway speed, whereas a machine learning model could predict the benefit of 'exporting' clinical decision making from one hospital to another, while allowing for differences in patient population between hospitals. By applying pathway simulation and machine learning together, we found a realistic ceiling of 15-25% use of thrombolysis across different hospitals and, in the seven hospitals studied, a realistic opportunity to double the number of patients with no significant disability that may be attributed to thrombolysis.

**Conclusions:** National clinical audit may be enhanced by a combination of pathway simulation and machine learning best allows for an understanding of key levers for improvement in hyper-acute stroke pathways, allowing for differences between local patient populations. These models, based on standard clinical audit data may be applied at scale while providing results at individual hospital level. The models facilitate understanding of variation, and levers for improvement, in stroke pathways, and help set realistic targets tailored to local populations.


## Strengths and limitations of this study

- The pathway simulation and methodology presented enhances the national clinical audit by providing insight into the inter-hospital variation in use and speed of thrombolysis.
- The methods allow reasonable thrombolysis targets to be set that are tailored to local stroke patient population characteristics.
- The methods (with code published in full) may be applied using Open Source Software, and may be applied at scale while providing tailored insight for individual hospitals.
- The methods are limited to information gathered in the national clinical audit for stroke.

For peer review only

# Introduction

NHS England describe clinical audit as a way of identifying whether healthcare is being provided in accordance with agreed standards, and where improvements could be made to improve outcomes for patients [1]. Audits may be local or national. In England the National Quality Improvement Programmes (HQIP), on behalf of the NHS, is responsible for overseeing and commissioning more than 30 clinical audits which form the National Clinical Audit Programme [2]. These collect and analyse data supplied by local clinicians.

The National Audit covering stroke is the Sentinel Stroke National Audit Programme (SSNAP) [3] . Stroke is a leading cause of death and disability worldwide, with an estimated 5.9 million deaths and 33 million stroke survivors in 2010 [4]. In England, Wales and Northern Ireland 85,000 people are hospitalised with stroke each year [5], and stroke is ranked third as a cause of disability-adjusted life years in the UK over the last 25 years [6].

SSNAP collects longitudinal data on the processes and outcomes of stroke care up to six months post stroke for more than 90% of stroke admissions to acute hospitals in England, Wales and Northern Ireland. Every year data from approximately 85,000 patients is collected. SSNAP publishes quarterly and yearly analysis of results on its website [3].

SSNAP audit data is used for a wide range of research such as investigating how type of stroke effects clinical decisions [7], how socio-economic factors influence risk of stroke, care received, and outcomes [8] , and how care processes may vary by time of day and day of week [9].

In this paper we report on the potential of using simulation and machine learning to enhance the output of the SSNAP clinical audits. In particular we focus on the acute stroke pathway and clinical decision making leading to the use of thrombolysis for the treatment of acute stroke, the only licensed drug treatment for acute stroke, and one that is critically time-dependent [10] with little or no benefit after 4.5 hours from stroke onset. The population benefit from thrombolysis has been limited by slow uptake of the treatment, and in-hospital delays to the administration of thrombolysis [11–13].

In England, Wales and Northern Ireland 11.1% of confirmed acute stroke patients receive thrombolysis, but use in individual acutely admitting stroke teams varies from 0 to 24.5% [5]. The lowest 10% of acutely admitting stroke teams administer thrombolysis to fewer than 5.9% of patients, whereas the top 10% administer thrombolysis to more than 16.7%. Time from arrival to thrombolysis (‘door-to-needle’) also varies significantly. The fastest 10% of hospitals have door-to-needle times of 40 minutes or less, whereas the slowest 10% have door-to-needle times of 85 minutes or more[5]. There is therefore considerable variation between hospitals in the use, and speed, of thrombolysis for acute stroke patients.

Analysis of patient pathway data coupled with computer simulation has previously allowed investigation and improvement of thrombolysis use in individual hospitals - increasing both the number of patients treated and reducing door-to-needle times [14,15]. These models have usually focused on the speed of the acute stroke pathway from arrival at hospital to giving thrombolysis, and have been tailored to use data available at an individual hospital [14].

Pathway modelling based on simulating process steps allows for good simulation of the speed of the stroke pathway, but cannot easily model differences in clinical decision making. We were interested in testing whether a model could dissect out the variation in thrombolysis rate that is dependent

For peer review only

upon differences in patient populations (e.g. age or stroke severity) in different hospitals, from the differences that are dependent on the culture of decision making at different hospitals (e.g. more cautious vs more aggressive clinical decision making). A variety of machine learning techniques now exist [16], which are able to make good predictions on pre-existing multidimensional data over a binary or categorical outcome variable (such as whether a patient receives thrombolysis or not). These have the potential to add modelling of clinical decision making to a model of the acute stroke pathway, with the aim of predicting what decision (to thrombolyse or not) would be made for the same patient in different hospitals. Models may also be trained on a reference standard set of hospitals (regarded as centres of clinical excellence) and use of thrombolysis for any patient predicted using that 'benchmark clinical decision making model'.

The aim of our work was to extend previous work on stroke thrombolysis pathway simulation in three significant ways: 1) to create a generic stroke thrombolysis pathway simulation model that could be easily applied to all hospitals in SSNAP, 2) extend the analysis to include factors other than door-to-needle times, with special focus on differences in clinical decision making as analysed and modelled with machine learning techniques, and 3) develop modelling framework that is open source and fast enough to run routine analysis at national level.

# Data

SSNAP data was obtained from 7 hospitals in England, for patients with a confirmed stroke over a period of two years (2013-2014) for each hospital. These data were secondary data, collected during routine care. No patient identifiable information was obtained.

For the pathway simulation model, the dataset contained 7,864 patient records with complete data for 12 parameters regarding their characteristics and time stamped pathway location. These data represent out-of-hospital onset of stroke (which account for 94% of all admissions recorded in the SSNAP data used).

For machine learning, only those patients with a completed NIH Stroke Scale and who had at least 30 minutes left to give thrombolysis were used (1,862 patients). As a precaution to maintain complete patient anonymity 17 patients aged under 40 had their age censored to 40, and 6 patients over the age of 100 had their age censored to 100.

# Pathway simulation model

The pathway simulation model is coded in Python/NumPy and is based on sampling from distributions based on real-world data.

Model parameters were set for each hospital by analysing anonymous data retrieved from SSNAP (Sentinel Stroke National Audit Programme). The model parameters, distribution types, and the values used are given in the online appendix.

For a patient to receive thrombolysis they must meet the following criteria: 1) stroke onset time known, 2) arrival at hospital within 4 hours of stroke onset, 3) have an ischaemic stroke and judged to be eligible for thrombolysis, and 4) be within the allowed thrombolysis time window (4.5 hours and 3 hours onset-to-treatment time for patients aged under 80, and 80+, respectively), when summing the process step times in the model. If a patient receives thrombolysis in the model then the probability of an additional good outcome (Modified Rankin Scale 0-1, no significant disability and able to carry out all usual activities) due to use of thrombolysis is calculated from the onset-to-

For peer review only

treatment time and is based on the meta-analysis by Emberson et al. [10]

## Clinical decision model (machine learning)

The clinical decision model aims to replicate the decision to give, or not give, treatment with thrombolysis for any given patient at any given hospital.

The model is coded in Python using the SciKit Learn [17] machine learning library.

The model predicts whether an individual patient would receive thrombolysis or not from a set of 50 parameters defining the patient's characteristics, clinical well-being, and hospital attended. Following a comparison of methods, a Random Forests method was chosen (see online appendix for a list of all features used, and a comparison of different machine-learning models).

This model is intended to make decisions based only on clinical presentation, assuming that there is time to give thrombolysis. Patients were included if they had been scanned with 30 minutes left to give thrombolysis (allowing 4.5 hours and 3 hours from onset to treatment for patients aged under 80, and 80+, respectively).

# Results

## Pathway simulation

The model was validated by comparing modelled (predicted) use of thrombolysis with actual use of thrombolysis. Actual use of thrombolysis was based on modelling a one year period, with replicates of 100 runs (each with different random number seeds) in order to determine expected year-to-year variation. The model showed very good correlation (R-square 0.981) between actual and predicted values though the model slightly under-predicted actual thrombolysis use by an average of 0.84% (figure 2).

The model was run with various 'what-if?' scenarios for each of the seven hospitals (Table 1):

1. Base case: Model based on parameters derived from current hospital-specific performance
2. Scenario A: Door-to-needle time fixed at 30 minutes (by fixing arrival-to-scan time and scan-to-thrombolysis both at 15 minutes (with no variation in either time)
3. Scenario B: 60% of ischaemic stroke patients scanned with 30 minutes left to treat receive thrombolysis (An analysis of ECASS-3/IST-3 results concluded that 591 out of 992, or 60%, of ischaemic stroke patients arriving within 4 hours of stroke onset were suitable for thrombolysis [18]).
4. Scenario C: Onset time known fixed at 77% (national SSNAP upper quartile for year 2015/16 [5])
5. Combination of above

For peer review only

*Table 1: Predicted thrombolysis use and clinical benefit across all modelled hospitals (1 to 7). Data shows: (Base) Model based on parameters derived from current performance; (A) Arrival-to-scan and scan-to-thrombolysis both fixed at 15 min (with no variation in either time); (B) Judged to be eligible for thrombolysis fixed at 60%; (C) Onset time known fixed at 77%; Combinations of the above. Results show mean and ±95% confidence limits (100 runs).*

| | | Scenarios | | | | | | | |
|---|---|---|---|---|---|---|---|---|---|
| | Hosp | Base | A | B | C | AB | AC | CB | ABC |
| Thrombo-lysis use (%) | 1 | 12.72 (0.08) | 19.46 (0.10) | 13.43 (0.09) | 14.41 (0.09) | 20.54 (0.10) | 22.05 (0.14) | 15.27 (0.12) | 23.38 (0.14) |
| | 2 | 6.97 (0.06) | 11.42 (0.08) | 10.77 (0.08) | 10.04 (0.08) | 17.45 (0.10) | 16.43 (0.09) | 15.50 (0.08) | 25.24 (0.10) |
| | 3 | 7.77 (0.06) | 11.13 (0.07) | 11.62 (0.07) | 11.24 (0.07) | 16.85 (0.08) | 15.77 (0.09) | 15.94 (0.09) | 23.97 (0.11) |
| | 4 | 6.37 (0.06) | 7.39 (0.06) | 10.94 (0.07) | 11.31 (0.08) | 12.76 (0.08) | 13.17 (0.09) | 19.35 (0.10) | 22.42 (0.11) |
| | 5 | 7.58 (0.09) | 11.19 (0.11) | 9.95 (0.11) | 12.99 (0.12) | 14.90 (0.13) | 19.56 (0.08) | 17.52 (0.08) | 25.86 (0.10) |
| | 6 | 13.42 (0.11) | 19.41 (0.13) | 15.97 (0.12) | 14.22 (0.12) | 23.24 (0.14) | 20.54 (0.10) | 16.99 (0.09) | 24.56 (0.10) |
| | 7 | 8.25 (0.06) | 10.66 (0.07) | 13.91 (0.08) | 11.21 (0.08) | 18.13 (0.09) | 14.58 (0.13) | 19.48 (0.14) | 25.05 (0.15) |
| Additional good outcomes per 1,000 patients | 1 | 11.11 (0.07) | 18.95 (0.11) | 11.74 (0.08) | 12.59 (0.08) | 20.00 (0.10) | 21.37 (0.14) | 13.29 (0.11) | 22.67 (0.14) |
| | 2 | 6.00 (0.06) | 11.35 (0.09) | 9.27 (0.07) | 8.67 (0.07) | 17.30 (0.10) | 16.48 (0.09) | 13.47 (0.07) | 25.35 (0.11) |
| | 3 | 6.39 (0.05) | 11.27 (0.07) | 9.56 (0.06) | 9.26 (0.06) | 17.04 (0.09) | 15.35 (0.09) | 12.73 (0.08) | 23.30 (0.11) |
| | 4 | 5.55 (0.05) | 7.15 (0.06) | 9.50 (0.07) | 9.83 (0.07) | 12.36 (0.08) | 12.66 (0.09) | 16.70 (0.09) | 21.55 (0.11) |
| | 5 | 6.61 (0.09) | 11.55 (0.12) | 8.68 (0.10) | 11.34 (0.11) | 15.39 (0.14) | 20.61 (0.09) | 15.51 (0.08) | 27.26 (0.11) |
| | 6 | 11.44 (0.10) | 18.98 (0.14) | 13.62 (0.11) | 12.10 (0.10) | 22.72 (0.15) | 20.14 (0.10) | 14.55 (0.08) | 24.12 (0.10) |
| | 7 | 7.35 (0.06) | 10.77 (0.08) | 12.42 (0.07) | 9.99 (0.07) | 18.30 (0.09) | 14.92 (0.14) | 17.54 (0.13) | 25.65 (0.17) |

For peer review only

In order to achieve the greatest improvement in thrombolysis use in each of the seven hospitals, for three hospitals (hospitals 1, 2 and 6) it would be best to improve the speed of the pathway, for two hospitals (hospitals 4 and 5) it would be best to improve determination of stroke onset time, and for two (hospitals 3 and 7) it would be best to judge more patients as eligible for thrombolysis for those scanned with time left to treat. If a priority is to maximise clinical outcome then for five hospitals

(hospitals 1, 2, 3, 5 and 6) it would be best to improve the speed of the pathway, for one hospital (hospital 4) it would be best to improve the determination of stroke onset time and for one (hospital 7) it would be best to judge more patients as eligible for thrombolysis for those scanned with time left to treat. Combining all changes in the model could produce thrombolysis rates up to 22-26%, and 23-26 additional non-disabled outcomes per 1,000 stroke patients admitted.

In the case of hospital 5, arrival-to-scan times could be *slowed* by an average of 30 minutes and clinical outcomes would still be greater if that hospital achieved a proportion of known stroke onset time equal to the national average.

## Clinical decision (machine learning) model

A machine learning model may be trained on a subset of patients to investigate how the difference in thrombolysis use between hospitals may be proportionally attributed to either the hospital or the local patient population. Table 2 shows the predicted use of thrombolysis in a set of patients that attend one hospital, based on decisions made from training at another hospital. Taking hospital 7 as an example, between 31% to 45% of the patients that currently attend hospital 7 (with time left to receive thrombolysis) might receive thrombolysis depending on which hospital decision making is used to train the model. Patient cohort also effects the predicted thrombolysis uses.  Taking hospital 7 as an example again, if the model is trained on decisions made for patients attending hospital 7, and different hospital patient groups are then analysed in the model then thrombolysis use is to be between 23% and 50% depending on the hospital patient group analysed.

For peer review only

*Table 2. Predicted thrombolysis use (for patients with patients scanned with time left to thrombolysis) if the decision to give thrombolysis is based on decisions made by a Random Forest model trained at different hospitals. The columns therefore represent the likely difference in thrombolysis use due to differences in decision making.*

*Actual thrombolysis use by hospital*

| 1 | 2 | 3 | 4 | 5 | 6 | 7 | All |
|---|---|---|---|---|---|---|---|
| 52% | 35% | 48% | 33% | 49% | 44% | 31% | 40% |

*Predicted thrombolysis use*

| | | Hospital patients actually attended | | | | | | | |
|---|---|---|---|---|---|---|---|---|---|
| | | 1 | 2 | 3 | 4 | 5 | 6 | 7 | All |
| Hospital used to train model | 1 | 52% | 42% | 58% | 50% | 67% | 57% | 45% | 51% |
| | 2 | 48% | 35% | 55% | 36% | 46% | 37% | 29% | 40% |
| | 3 | 53% | 38% | 48% | 46% | 58% | 41% | 34% | 44% |
| | 4 | 40% | 28% | 48% | 33% | 52% | 29% | 26% | 35% |
| | 5 | 50% | 36% | 50% | 40% | 49% | 45% | 37% | 43% |
| | 6 | 49% | 32% | 55% | 44% | 59% | 44% | 39% | 44% |
| | 7 | 42% | 23% | 42% | 31% | 50% | 36% | 31% | 35% |

For peer review only

## Combining pathway simulation and machine learning

The output from machine learning may be incorporated into the stroke pathway model by using the machine learning model to make the decision in the pathway model about whether a patient is 'judged to be eligible for thrombolysis (for patients scanned with 30 minutes left to administer thrombolysis)'. This should tailor the clinical decision to the local population, without being affected by any particular hospital's predisposition to use thrombolysis. The 'judged to be eligible for thrombolysis' parameter in the pathway model may take its value from a machine learning model trained using a reference set of hospitals. The clinical decision making from these reference hospitals may be used to predict which of the patients from the hospital under study are eligible for thrombolysis.

Table 3 compares base case hospital performance (predicted thrombolysis use and clinical benefit) with the performance obtainable by a new realistic 'alternative' practice which is in part informed by the Random Forest machine learning model: (1) the proportion of patients with a known stroke onset time is set at the national median (67%) unless a hospital is already higher, (2) the door-to-needle time is set to 40 minutes for 90% of patients (20 minutes arrival-to-scan, and 20 minutes scan-to-needle) with the other 10% of patients not receiving a scan within 4 hours of arrival, and (3) the clinical decision to administer thrombolysis for those patients scanned with 30 minutes left to

treat is set by the machine learning model trained from a reference hospital (this example uses the hospital that has the highest use of thrombolysis for those patients scanned with time to treat). Resulting thrombolysis targets vary from 16-25% depending on the hospital (base case 6 to 13%).

For peer review only

*Table 3. Combining pathway simulation and machine learning. Predicted thrombolysis use and clinical benefit (additional good outcomes per 1,000 admitted patients) across all modelled hospitals (1 to 7) from the pathway simulation. Data shows: (Base) Model based on parameters derived from current performance; Alternative 'realistic target' settings, fixing the proportion of known stroke onset times to the national SSNAP average (67% median) unless the hospital currently performs higher, fixing arrival-to-scan and scan-to-needle to 20 minutes each (with 10% of patients not scanned within 4 hours), and fixing the proportion of treatable patients (scanned with 30 minutes left to treat) according to the output of the machine learning model based on the hospital with the maximum predicted proportion given thrombolysis. Data shows mean and 95% confidence intervals.*

| | Thrombolysis use (%) | | Clinical benefit: Additional good outcomes per 1,000 admissions | |
|---|---|---|---|---|
| Hospital | Current | Alternative | Current | Alternative |
| 1 | 13.0 (0.3) | 18.8 (0.3) | 11.4 (0.3) | 17.6 (0.3) |
| 2 | 6.8 (0.2) | 15.6 (0.3) | 5.9 (0.2) | 14.9 (0.3) |
| 3 | 7.9 (0.2) | 21.7 (0.3) | 6.5 (0.1) | 21.2 (0.3) |
| 4 | 6.4 (0.2) | 17.1 (0.3) | 5.5 (0.2) | 16.0 (0.3) |
| 5 | 7.6 (0.3) | 25.8 (0.5) | 6.7 (0.3) | 25.7 (0.5) |
| 6 | 13.1 (0.3) | 23.2 (0.4) | 11.3 (0.3) | 21.8 (0.4) |
| 7 | 8.2 (0.2) | 16.8 (0.3) | 7.2 (0.2) | 16.3 (0.3) |

# Discussion

While there is no agreed benchmark for the proportion of stroke patients who should receive thrombolysis, it has been suggested that about half of stroke patients, if they arrive at hospital in time, could be clinically suitable for thrombolysis [18]. In practice the greatest proportion of patients treated with thrombolysis in English hyper-acute stroke services is close to 20% [5]. These figures contrast with an average 12% across England and Wales [5], and a range of 6%-14% across the seven acute stroke centres in our study. Internationally thrombolysis rates also appear on average to be lower than best-practice centres: for example rates have been reported to have been 14.6% in the Netherlands in 2012 [19] and 5.4% in USA in 2010 [20] (for patients aged 65 years or more).

Our pathway simulation model slightly under-predicted actual use of thrombolysis. This is most likely due to the model applying a strict cut-off of allowable time from onset of stroke to giving thrombolysis. In a real clinical setting a little bit of flexibility may be applied.

As patients move through the stroke pathway, an unknown stroke onset time would be the first key barrier to thrombolysis in our model – a barrier to thrombolysis previously noted [21]. Though a qualitative analysis of methods for determining the onset time of a stroke was beyond the scope of this study, it was clear that hospitals differed in how this information was gathered, such as whether they relied on information from paramedics only, or whether the hospital clinician would also investigate an unknown stroke onset time. In our modelling study we looked at the potential impact

For peer review only

of reaching the national upper quartile for ascertaining stroke onset times. In one of the seven hospitals this factor was the single largest in attaining improvements to the thrombolysis rate. For patients with unknown time of onset, it may be possible to use advanced scanning methods to estimate stroke onset times [22] and further increase the population eligible for thrombolysis treatment.

Reductions to in-hospital treatment delays have been the focus of previous modelling approaches [14,15], and the 'need for speed' has frequently been stressed [11,12]. Our study found that compared with current state it was reasonable to expect that improvements to pathway speed and reliability could achieve close to 50% increase in the number of disability free patients. Our modelling was based on consistently achieving 30 minutes door-to-needle times. It may be possible to be even more aggressive on process time as speeds of 20 minutes door-to-needle times have been reported [23]. Those hospitals in our study where paramedics took FAST-positive patients straight to the scanner (bypassing ED) had significantly faster arrival-to-scan times, and this may be a more widely applicable approach, although it was not responsible on its own for a higher thrombolysis rate in those centres, illustrating the multi-factorial influences on both thrombolysis rate and door-to-needle time; it may sometimes be best to accept slower pathway speeds in order to improve other factors in the pathway (such as ascertaining stroke onset time). A challenge for smaller hospitals is to have consistently fast processes 24 hours per day seven days per week; scanners must be rapidly available for stroke patients at any time of day and day of week.

The final hurdle investigated was in the confidence in using thrombolysis for patients scanned in time. Use of thrombolysis, when time allowed, varied significantly between hospitals. Physician uncertainty, or lack of confidence in thrombolysis, has previously been identified as a barrier to use of thrombolysis [24]. Despite meta-analysis demonstrating the clinical benefit of thrombolysis [10], the use and benefit of thrombolysis has still recently been under scrutiny [25]. This could certainly explain the large between-hospital variation in thrombolysis rates we observed for those patients scanned with time left to receive thrombolysis.

Scenarios modelled using our stand-alone pathway model used a standard 'optimum' rate for the clinical decision to use thrombolysis based on an analysis of the IST-3 trial. However this scenario assumes all hospitals receive patients who have similar characteristics overall to those in the IST-3 trial, and assumes that the IST-3 trial criteria should be a benchmark for all hospitals. The machine learning model allows for differences in patient populations between hospitals (for example reflecting different age demographics between different regions), and allows for differences in real-life (rather than clinical trial) decision making. Using results from the machine learning model, which focuses solely on clinical suitability for thrombolysis when there is time left to treat after the scan, enabled the pathway model to be refined and tailored further to local hospital populations. Machine learning models had an accuracy of 80-82% in predicting decisions made. The current performance, while falling short of performance necessary for real-world decision making or guidance, is still useful in audit to understand variation in use of thrombolysis,

Using machine learning algorithms we found that differences in thrombolysis rate, for patients with time left to thrombolyse after the scan, can be explained by differences in patient population and in differences in decision making between hospitals. Rather than being based on a set of simple rules, our machine learning model learn from the clinical decision in real-life settings. A machine learning model allows prediction of potential thrombolysis use given the characteristics of the local population, but based on real-life clinical decision making at a range of hospitals, identifying those

For peer review only

hospitals which appear to be outliers in their decision making. Bembernek et al.[18] have suggested that if the inclusion criteria of the IST-3 trial are used to determine suitable for thrombolysis, about 60% of ischaemic stroke patients (equivalent to 50% all stroke patients, if 85% of patients have ischaemic stroke could be suitable for thrombolytic treatment) if they arrive in time and the pathway is efficient. This overall figure however does not allow for differences in patient mix in different geographic areas. A clinician reviewing their own use of thrombolysis may conclude that their use is different because their patients are different. Machine learning confirms differences in the patients populations, but also suggests differences in clinical decision making. By applying machine learning, feedback may be given regarding their use of thrombolysis compared with other hospitals allowing for differences in local populations. This may be especially useful if a benchmark group of hospitals (acknowledged centres of excellence in stroke care) is used the train the model.

When applying 'stretch targets' (e.g. basing overall use of thrombolysis on an analysis of the IST-3 trial) to stroke pathways in the pathway model we found that maximum thrombolysis use could be about 22-26% of all emergency confirmed stroke admissions. This could have the benefit of producing another 22-27 people with no significant disability for every 1,000 confirmed stroke patients admitted, with potential to also improve outcomes for those who cannot be classified as having no significant disability even after use of thrombolysis. Using slightly less challenging targets for the acute stroke pathway process, and using machine learning to mimic decisions to thrombolyse adjusted for local populations we found that individual hospital targets could realistically be set in the 16-25% range, which would lead to another 16-25 people with no significant disability for every 1,000 patients admitted. The upper limit varied between hospitals based on the patient mix attending that hospital. Such results warn against dangers of setting any universal expectation of use of thrombolysis

This approach has the potential to be applied locally or, as it is based solely on SSNAP data, as part of the national audit of stroke services. The methods have the potential to be automated, allowing for incorporation into the quarterly stroke service audit conducted by SSNAP. Where clinical decision making appears to be significantly different from a reference group of hospital the machine learning model may be used to identify a small group of patients for review, where clinical decision making appears to be different from a model trained on an agreed benchmark set of hospitals.

Our work is not the first work to apply simulation or machine learning in stroke. Previous work has been published on using pathway simulation at an individual hospital level [14,15]. We build on previous work by using standardised data and an open simulation framework to enable pathway simulation to be performed at scale across all hospital covered by the national stroke audit. Previous work has also been published using machine learning to predict risk of stroke [26], and likely outcome [27–29]. where our work adds novelty is firstly in the combination with pathway simulation, and the use of machine learning to investigate differences in clinical decision making between hospitals, and to apply "what if?" scenario testing of what might the likely clinical benefit be of standardising clinical decision making in accordance with recognised centres of excellence, taking into account differences in local patient populations.

*Strengths and weakness*

A key strength of our study is that, since the models are based solely on SSNAP data, the models developed may be applied to all UK hospitals. The use of Open Source software and the provision

For peer review only

of our code in a public repository should also facilitate easier adoption. Monks et al. [30]have noted that when modeling stroke pathways the level of detail of the model should depend on the questions being addressed. In our approach we consciously limit ourselves to the detail found in SSNAP data. A weakness, therefore, is that the models as they are presented cannot address questions that require data outside of the SSNAP data set. Our models highlight the improvements that can be achieved by an overall pathway stage (such as arrival-to-scan) but do not inform how improvement in that step could/should be achieved.

A strength of our modelling compared with normal approaches to stroke pathway modelling [30] is that we have used machine learning in order to both allow for differences in local stroke patient populations (e.g. differences in age, gender, and severity), and to understand differences in decision making between hospitals. A limitation of this approach is that the modelling is limited to hospital level, and will not uncover differences in use of thrombolysis by different practitioners within the same hospital. Nevertheless, the model will pick up overall organisational cultural attitudes towards use of thrombolysis in stroke (such as whether one hospital is more or less likely to use thrombolysis for patients with less severe stroke).

The use of Open Source software and the provision of our code in a public repository should also facilitate easier adoption, or refinement of the approach by others.

In this study we have not focussed on how implementation of changes may be brought about in different hospitals. It is possible that some improvements in the pathway (such as rapid availability to scan 24/7) may only be possible by centralisation of services into larger units who may have more resources to deploy [31–33]. Our model will show the benefit of reducing pathway speed and variability, but does not imply such a change is necessary always possible in smaller hospitals.

## Conclusions

The stroke clinical audit reports on thrombolysis usage (percentage of patients receiving thrombolysis), and time to thrombolysis. Inter-hospital variation in use of thrombolysis may be due to (1) differences in stroke pathway, (2) differences in patient population characteristics, or (3) differences in clinical decision making. In our study we present a method that qualitatively analyses key components of the stroke pathway in a manner that may be applied at scale while producing individualised results for each hospital, highlighting how thrombolysis use and speed may best be improved. We believe this type of approach will augment current outputs from national clinical audits.

For peer review only

# References

1 NHS England. Clinical Audit. 2018.https://www.england.nhs.uk/clinaudit/ (accessed 5 Oct 2018).

2 National quality improvement programmes (HQIP). The National Clinical Audit Programme. https://www.hqip.org.uk/a-z-of-nca/ (accessed 5 Oct 2018).

3 Royal College Of Physians. Sentinel Stroke National Audit Programme (SSNAP): Web Site. https://www.strokeaudit.org/

4 Feigin VL, Forouzanfar MH, Krishnamurthi R, *et al.* Global and regional burden of stroke during 1990-2010: Findings from the Global Burden of Disease Study 2010. *The Lancet* 2014;**383**:245–255. doi:10.1016/S0140-6736(13)61953-4

5 Sentinel Stroke Audit Programme (SSNAP). *Apr2015Mar2016-AnnualResultsPortfolio*. 2016.

6 Newton JN, Briggs ADM, Murray CJL, *et al.* Changes in health in England, with analysis by English regions and areas of deprivation, 1990-2013: A systematic analysis for the Global Burden of Disease Study 2013. *The Lancet* 2015;**386**:2257–2274. doi:10.1016/S0140-6736(15)00195-6

7 Parry-Jones AR, Paley L, Bray BD, *et al.* Care-limiting decisions in acute stroke and association with survival: analyses of UK national quality register data. *Int J Stroke* 2016;**11**:321–31. doi:10.1177/1747493015620806

8 Bray BD, Paley L, Hoffman A, *et al.* Socioeconomic disparities in first stroke incidence, quality of care, and survival: a nationwide registry-based cohort study of 44 million adults in England. *Lancet Public Health* 2018;**3**:e185–93. doi:10.1016/S2468-2667(18)30030-6

9 Bray BD, Cloud GC, James MA, *et al.* Weekly variation in health-care quality by day and time of admission: a nationwide, registry-based, prospective cohort study of acute stroke care. *The Lancet* 2016;**388**:170–7. doi:10.1016/S0140-6736(16)30443-3

10 Emberson J, Lees KR, Lyden P, *et al.* Effect of treatment delay, age, and stroke severity on the effects of intravenous thrombolysis with alteplase for acute ischaemic stroke: A meta-analysis of individual patient data from randomised trials. *The Lancet* 2014;**384**:1929–1935. doi:10.1016/S0140-6736(14)60584-5

11 Meretoja A, Keshtkaran M, Saver JL, *et al.* Stroke thrombolysis: Save a minute, save a day. *Stroke* 2014;**45**:1053–1058. doi:10.1161/STROKEAHA.113.002910

12 Saver JL. Time is brain - Quantified. *Stroke* 2006;**37**:263–266. doi:10.1161/01.STR.0000196957.55928.ab

13 Lyden P. Thrombolytic Therapy for Acute Stroke — Not a Moment to Lose. *N Engl J Med* 2008;**359**:1393–1395. doi:10.1056/NEJMe0806335

14 Monks T, Pitt M, Stein K, *et al.* Maximizing the population benefit from thrombolysis in acute ischemic stroke: A modeling study of in-hospital delays. *Stroke* 2012;**43**:2706–2711. doi:10.1161/STROKEAHA.112.663187

15 Lahr M, van der Zee D-J, Luijckx G, *et al.* A simulation-based approach for improving

For peer review only

utilization of thrombolysis in acute brain infarction. *Med Care* 2013;**51**:1101–1105. doi:10.1097/MLR.0b013e3182a3e505

16 Kotsiantis SB, Zaharakis I, Pintelas P. Supervised machine learning: A review of classification techniques. *Informatica* 2007;**31**:249–268. doi:10.1115/1.1559160

17 Pedregosa F, Varoquaux G, Gramfort A, *et al.* Scikit-learn: Machine Learning in Python. *J Mach Learn Res* 2011;**12**:2825–30.

18 Bembenek J, Kobayashi A, Sandercock P, *et al. How many patients might receive thrombolytic therapy in the light of the ECASS-3 and IST-3 data?* 2010. doi:10.1111/j.1747-4949.2010.00479.x

19 Scherf S, Limburg M, Wimmers R, *et al.* Increase in national intravenous thrombolysis rates for ischaemic stroke between 2005 and 2012: is bigger better? *BMC Neurol* 2016;**16**:53. doi:10.1186/s12883-016-0574-7

20 George BP, Asemota AO, Dorsey ER, *et al.* United States trends in thrombolysis for older adults with acute ischemic stroke. *Clin Neurol Neurosurg* 2015;**139**:16–23. doi:10.1016/j.clineuro.2015.08.031

21 Aoki J, Kimura K, Iguchi Y, *et al.* FLAIR can estimate the onset time in acute ischemic stroke patients. *J Neurol Sci* 2010;**293**:39–44. doi:10.1016/j.jns.2010.03.011

22 Aoki J, Kimura K, Iguchi Y, *et al.* FLAIR can estimate the onset time in acute ischemic stroke patients. *J Neurol Sci* 2010;**293**:39–44. doi:10.1016/j.jns.2010.03.011

23 Meretoja A, Strbian D, Mustanoja S, *et al.* Reducing in-hospital delay to 20 minutes in stroke thrombolysis. *Neurology* 2012;**79**:306–313. doi:10.1212/WNL.0b013e31825d6011

24 Kwan J, Hand P, Sandercock P. A systematic review of barries to delivery of thrombolysis for acute stroke. *Age Ageing* 2004;**33**:116–121. doi:10.1093/ageing/afh064

25 Alper BS, Malone-Moses M, McLellan JS, *et al.* Thrombolysis in acute ischaemic stroke: time for a rethink? *Bmj* 2015;**350**:h1075–h1075. doi:10.1136/bmj.h1075

26 Khosla A, Cao Y, Lin CC-Y, *et al.* An integrated machine learning approach to stroke prediction. *Proc 16th ACM SIGKDD Int Conf Knowl Discov Data Min - KDD 10* 2010;:183. doi:10.1145/1835804.1835830

27 Rehme AK, Volz LJ, Feis D-L, *et al.* Identifying Neuroimaging Markers of Motor Disability in Acute Stroke by Machine Learning Techniques. *Cereb Cortex* 2015;**25**:3046–56. doi:10.1093/cercor/bhu100

28 Asadi H, Dowling R, Yan B, *et al.* Machine learning for outcome prediction of acute ischemic stroke post intra-arterial therapy. *PLoS ONE* 2014;**9**:14–19. doi:10.1371/journal.pone.0088225

29 Bentley P, Ganesalingam J, Carlton Jones AL, *et al.* Prediction of stroke thrombolysis outcome using CT brain machine learning. *NeuroImage Clin* 2014;**4**:635–40. doi:10.1016/j.nicl.2014.02.003

30 Monks T, van der Zee D-J, Lahr M, *et al.* A framework to accelerate simulation studies of hyperacute stroke systems. *Oper Res Health Care* 2017;**15**:57–67. doi:10.1016/j.orhc.2017.09.002

For peer review only

31 Lahr MMH, van der Zee D-J, Luijckx G-J, *et al.* Centralising and optimising decentralised stroke care systems: a simulation study on short-term costs and effects. *BMC Med Res Methodol* 2017;**17**:5. doi:10.1186/s12874-016-0275-3

32 Morris S, Hunter RM, Ramsay AIG, *et al.* Impact of centralising acute stroke services in English metropolitan areas on mortality and length of hospital stay: difference-in-differences analysis. *Bmj* 2014;**349**:g4757–g4757. doi:10.1136/bmj.g4757

33 Ramsay AIG, Morris S, Hoffman A, *et al.* Effects of centralizing acute stroke services on stroke care provision in two large metropolitan areas in England. *Stroke* 2015;**46**:2244–2251. doi:10.1161/STROKEAHA.115.009723

For peer review only

# Figure legends

*Figure 1. Modelled sequence of steps of the emergency stroke pathway leading to thrombolysis.*

*Figure 2. Validation of the pathway simulation model, comparing actual to modelled (predicted) thrombolysis use at seven hospital. Points show mean predicted thrombolysis use for all confirmed stroke patients arriving at hospital, with bars showing 5$^{th}$ and 95$^{th}$ percentiles from 100 runs, with each run modelling one year.*

For peer review only

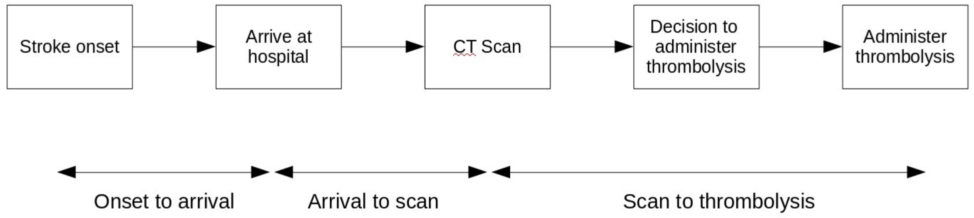


Figure 1. Modelled sequence of steps of the emergency stroke pathway leading to thrombolysis.

494x122mm (72 x 72 DPI)

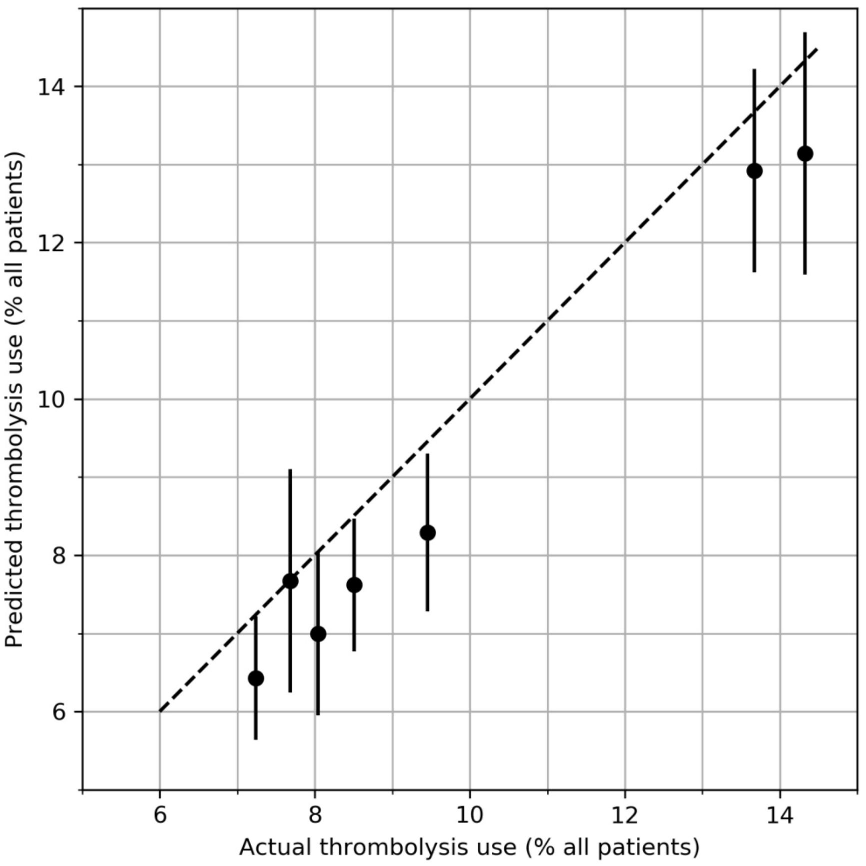


Figure 2. Validation of the pathway simulation model, comparing actual to modelled (predicted) thrombolysis use at seven hospital. Points show mean predicted thrombolysis use for all confirmed stroke patients arriving at hospital, with bars showing 5th and 95th percentiles from 100 runs, with each run modelling one year.

152x152mm (300 x 300 DPI)

# Enhancing clinical audit with pathway simulation and machine learning: Supplementary material

Michael Allen[1], Kerry Pearn[1], Thomas Monks[2], Benjamin Bray[3], Richard Everson[4], Andrew Salmon[1], Martin James[5] and Ken Stein[1]

[1]University of Exeter Medical School & NIHR Collaboration for Leadership in Applied Health Research and Care South West Peninsula.
[2]NIHR CLAHRC Wessex
[3]Sentinel Stroke National Audit Programme (SSNAP), Kings College, London
[4]Computer Sciences, University of Exeter
[5]Royal Devon and Exeter NHS Foundation Trust & NIHR Collaboration for Leadership in Applied Health Research and Care South West Peninsula.

October 2018

## Contents

**1 Hospital performance parameters used in the pathway simulation model** **2**

**2 Machine Learning Model** **4**

For peer review only

# 1 Hospital performance parameters used in the pathway simulation model

A description parameters used in the thrombolysis pathway simulation model, the distribution type used, and the range of values used in the base case models are shown in table 1. Note that process times use log (ln) normal distributions. Table 2 shows individual parameters for each of seven hospitals; these were derived from SSNAP data.

Table 1: Pathway simulation paraemters

| Parameter | Distribution | Range used (base case) |
|---|---|---|
| 1. Allowed onset-to-treatment time for age up to 80 (mins) | Fixed | 270 |
| 2. Allowed onset-to-treatment time for age 80 +(mins) | Fixed | 180 |
| 3. Aged 80+ | Bernoulli | 0.413-0.513 |
| 4. Arrivals per year | Fixed | 300-800 |
| 5. Onset time known | Bernoulli | 0.434-0.727 |
| 6. Onset-to-arrival <4 hours | Bernoulli | 0.677-0.705 |
| 7. Onset-to-arrival time (mins) | Log(ln) normal | Mean (ln): 4.411-4.609<br>StdDev (ln): 0.475-0.588 |
| 8. Arrival-to-scan <4hrs | Bernoulli | 0.78-0.97 |
| 9. Arrival-to-scan time (mins) | Log (ln) normal | Mean (ln): 2.477-3.776<br>StdDev (ln): 0.750-1.271 |
| 10. Ischaemic stroke | Bernoulli | 0.85-0.89 |
| 11. Eligible for thrombolysis | Bernoulli | 0.344-0.572 |
| 12. Scan-to-thrombolysis (mins) | Log (ln) normal | Mean (ln): 2.970-3.791<br>StdDev (ln): 0.578-0.849 |

Notes:

1. The allowed onset-to-treatment time for patients aged up to 80 is 4.5 hours.
2. The allowed onset-to-treatment time for patients aged 80+ is 3 hours.
3. The proportion of patients aged 80+ varies between hospitals.
4. The number of arrivals is the number of confirmed stroke patients in SSNAP per year
5. Onset time known is binary yes/no. Known onset time may be recorded as precise or estimated in SSNAP. Those without a known onset time cannot be treated with thrombolysis in the model.
6. Onset to arrival <4hrs is the proportion of patients with known stroke onset that arrive within 4 hours of onset. Those arriving more than 4 hours after onset cannot be treated with thrombolysis in the model.
7. Onset to arrival time is a log-normal distribution. It is applied only to those patients arriving within 4 hours of known stroke onset.
8. Arrival to scan time <4hrs is the proportion of patients (those with known stroke onset time and arriving within 4 hours of onset) that receive a CT head scan within 4 hours of arrival.
9. Arrival to scan time is a log-normal distribution. It is applied only to those patients arriving within 4 hours of known stroke onset, and receiving a scan within 4 hours of arrival.
10. Ischaemic stroke is the proportion of patients with ischaemic (rather than haemorrhagic stroke). It is applied only to those patients arriving within 4 hours of known stroke onset, and receiving a scan within 4 hours of arrival.
11. Eligible for thrombolysis is the proportion of ischaemic stroke patients (arriving within 4 hours of known stroke onset, and scanned within 4 hours of arrival) who are considered clinically eligible for thrombolysis. This figure is obtained by examining the proportion of ischaemic stroke patients who are scanned with at least 30 minutes left to thrombolyse who are given thrombolysis.
12. Scan to thrombolysis is a log-normal distribution. It is applied only to those patients arriving within 4 hours of known stroke onset, receiving a scan within 4 hours of arrival, and are ischaemic strokes considered eligible for thrombolysis.


For peer review only - http://bmjopen.bmj.com/site/about/guidelines.xhtml

Table 2: Pathway simulation parameters for individual hospitals

| Hospital | 1 | 2 | 3 | 4 | 5 | 6 | 7 |
|---|---|---|---|---|---|---|---|
| Arrivals per year | 600 | 600 | 800 | 700 | 300 | 350 | 700 |
| Aged 80 | 0.502 | 0.513 | 0.413 | 0.498 | 0.458 | 0.511 | 0.489 |
| Onset time known | 0.678 | 0.537 | 0.533 | 0.434 | 0.446 | 0.727 | 0.562 |
| Onset-to-arrival < 4 hours | 0.684 | 0.698 | 0.693 | 0.677 | 0.697 | 0.705 | 0.703 |
| Onset-to-arrival time (mean, ln) | 4.568 | 4.471 | 4.557 | 4.609 | 4.411 | 4.527 | 4.463 |
| Onset-to-arrival time (StdDev) | 0.513 | 0.539 | 0.475 | 0.519 | 0.558 | 0.493 | 0.588 |
| Arrival-to-scan < 4 hours | 0.844 | 0.783 | 0.947 | 0.979 | 0.837 | 0.849 | 0.913 |
| Arrival-to-scan (mean, ln) | 3.645 | 3.421 | 3.776 | 2.477 | 3.702 | 3.352 | 3.097 |
| Arrival-to-scan (StdDev, ln) | 0.905 | 1.097 | 0.750 | 1.271 | 0.814 | 0.974 | 0.997 |
| Ischaemic stroke | 0.864 | 0.885 | 0.852 | 0.858 | 0.892 | 0.857 | 0.882 |
| Eligible for thrombolysis | 0.557 | 0.386 | 0.392 | 0.344 | 0.445 | 0.495 | 0.347 |
| Scan-to-thrombolysis (mean, ln) | 3.084 | 3.589 | 3.791 | 3.676 | 3.540 | 3.565 | 3.580 |
| Scan-to-thrombolysis (StdDev, ln) | 0.849 | 0.635 | 0.585 | 0.578 | 0.616 | 0.594 | 0.726 |

The model runs 100 trials simulating 100 different years, with final results recording mean and variation between years. The outputs of the pathway model are:

1. Baseline good outcomes (median)
2. Baseline good outcomes per 1000 patients ($5^{th}$ percentile)
3. Baseline good outcomes per 1000 patients ($95^{th}$ percentile)
4. Baseline good outcomes per 1000 patients (mean)
5. Baseline good outcomes per 1000 patients (stdev)
6. Baseline good outcomes per 1000 patients (95% confidence interval)
7. Percent Thrombolysis (median)
8. Percent Thrombolysis ($5^{th}$ percentile)
9. Percent Thrombolysis ($95^{th}$ percentile)
10. Percent Thrombolysis (mean)
11. Percent Thrombolysis (stdev)
12. Percent Thrombolysis (95% confidence interval)
13. Additional good outcomes per 1000 patients (median)
14. Additional good outcomes per 1000 patients ($5^{th}$ percentile)
15. Additional good outcomes per 1000 patients ($95^{th}$ percentile)
16. Additional good outcomes per 1000 patients (mean)
17. Additional good outcomes per 1000 patients (stdev)
18. Additional good outcomes per 1000 patients (95% confidence interval)

For peer review only

## 2 Machine Learning Model

Table 3 shows the features used in the machine learning models. These features were all obtained from SSNAP data.

Table 3: Features used in the machine learning models

| Feature | Type | Feature | Type |
|---|---|---|---|
| Hosp 1 | Binary | Anticoag before stroke 1 | Binary |
| Hosp 2 | Binary | Anticoag before stroke NK | Binary |
| Hosp 3 | Binary | Stroke severity group 1. No stroke symptoms | Binary |
| Hosp 4 | Binary | Stroke severity group 2. Minor | Binary |
| Hosp 5 | Binary | Stroke severity group 3. Moderate | Binary |
| Hosp 6 | Binary | Stroke severity group 4. Moderate to severe | Binary |
| Hosp 7 | Binary | Stroke severity group 5. Severe | Binary |
| Male | Binary | Stroke Type I | Binary |
| Age (years) | Integer | Stroke Type PIH | Binary |
| 80 | Binary | S2RankinBeforeStroke | Integer |
| Onset Time Known Type BE | Binary | S2NihssArrival | Integer |
| Onset Time Known Type NK | Binary | S2NihssArrivalLocQuestions | Integer |
| Onset Time Known Type P | Binary | S2NihssArrivalLocCommands | Integer |
| # Comorbidities | Integer | S2NihssArrivalBestGaze | Integer |
| 2+ comorbidotes | Binary | S2NihssArrivalVisual | Integer |
| Congestive HF | Binary | S2NihssArrivalFacialPalsy | Integer |
| Hypertension | Binary | S2NihssArrivalMotorArmLeft | Integer |
| Atrial Fib | Binary | S2NihssArrivalMotorArmRight | Integer |
| Diabetes | Binary | S2NihssArrivalMotorLegLeft | Integer |
| TIA | Binary | S2NihssArrivalMotorLegRight | Integer |
| Co-mordity | Binary | S2NihssArrivalLimbAtaxia | Integer |
| Antiplatelet 0 | Binary | S2NihssArrivalSensory | Integer |
| Antiplatelet 1 | Binary | S2NihssArrivalBestLanguage | Integer |
| Antiplatelet NK | Binary | S2NihssArrivalDysarthria | Integer |
| Anticoag before stroke 0 | Binary | S2NihssArrivalExtinctionInattention | Integer |

Table 4 shows performance of different machine learning models (Support Vector Machines models were preformed with linear or radial basis function kernels). All models used the SciKit Learn library for Python. The Random Forests model was chosen for the work described in the paper.

The models were evaluated for:

- *accuracy*: The proportion of thrombolysis decisions correctly predicted.
- *sensitivity*: The proportion of positive thrombolysis decisions correctly predicted.
- *specificity*: The proportion of negative thrombolysis decisions correctly predicted.
- *ROC area*: The area of the operator-receiver curve.

Table 4: Performance of machine learning models

| Model | accuracy | sensitivity | specificity | ROC area |
|---|---|---|---|---|
| Logistic regression | 0.807 (0.010) | 0.788 (0.015) | 0.819 (0.012) | 0.882 (0.005) |
| Random Forest | 0.819 (0.010) | 0.792 (0.017) | 0.837 (0.011) | 0.889 (0.007) |
| SVM (linear) | 0.807 (0.008) | 0.820 (0.012) | 0.798 (0.012) | 0.880 (0.005) |
| SVM (rbf) | 0.813 (0.006) | 0.820 (0.013) | 0.808 (0.008) | 0.880 (0.006) |
| Neural Network | 0.807 (0.010) | 0.794 (0.017) | 0.816 (0.012) | 0.882 (0.005) |

For peer review only

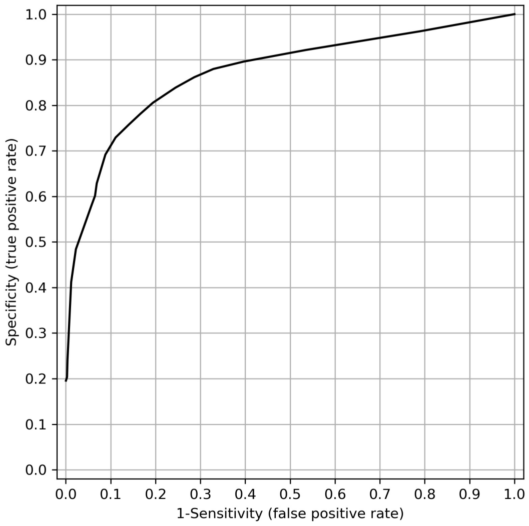


Figure 1: Receiver Operator Curve for the Random Forest model

Figure 1 shows the Receiver Operator Curve for the Random Forest model. It is possible to have 80% sensitivity and specificity simultaneously.

For peer review only



For peer review only - http://bmjopen.bmj.com/site/about/guidelines.xhtml

BMJ Open

# Can clinical audits be enhanced by pathway simulation and machine learning? An example from the acute stroke pathway.

| | |
|---|---|
| Journal: | *BMJ Open* |
| Manuscript ID | bmjopen-2018-028296.R1 |
| Article Type: | Research |
| Date Submitted by the Author: | 05-Aug-2019 |
| Complete List of Authors: | Allen, Michael; University of Exeter, Medical School<br>Pearn, Kerry; University of Exeter, Medical School<br>Monks, T; University of Southampton<br>Bray, Benjamin D.; Royal Coll Physicians<br>Everson, Richard; University of Exeter, Computer Sciences<br>Salmon, Andrew; University of Exeter<br>James, Martin; Royal Devon & Exeter NHS trust<br>Stein, Ken; University of Exeter Medical School, PenTAG |
| <b>Primary Subject Heading</b>: | Health services research |
| Secondary Subject Heading: | Cardiovascular medicine |
| Keywords: | Stroke < NEUROLOGY, thrombolysis, alteplase, simulation, machine learning, health services research |
| | |

SCHOLARONE™
Manuscripts

# Can clinical audits be enhanced by pathway simulation and machine learning? An example from the acute stroke pathway.

Michael Allen*, Honorary Research Fellow, University of Exeter Medical School, St Luke's Campus, Heavitree Road, EXETER, EX1 2LU, UK

Kerry Pearn, Associate Research Fellow, University of Exeter Medical School, St Luke's Campus, Heavitree Road, EXETER, EX1 2LU, UK

Thomas Monks, Principal Research Fellow, NIHR CLAHRC Wessex Data Science Hub, Room AA72 South Academic Block, Southampton General Hospital, SOUTHAMPTON, SO16 6YD

Benjamin Bray, Research Director, Sentinel Stroke National Audit Programme (SSNAP), Kings College, London

Richard Everson, Professor of Machine Learning, Computer Sciences, University of Exeter

Andrew Salmon, Associate Research Fellow, University of Exeter Medical School, St Luke's Campus, Heavitree Road, EXETER, EX1 2LU, UK

Martin James, Consultant Stroke Physician, Royal Devon & Exeter NHS trust, Barrack Road, EXETER, EX2 5DW, UK

Ken Stein, Deputy Director of PenCLAHRC, University of Exeter Medical School, St Luke's Campus, Heavitree Road, EXETER, EX1 2LU, UK

**keywords**: stroke, thrombolysis, alteplase, simulation, machine learning

*Correspondence to: m.allen@exeter.ac.uk

The Corresponding Author has the right to grant on behalf of all authors and does grant on behalf of all authors, a worldwide licence to the Publishers and its licensees in perpetuity, in all forms, formats and media (whether known now or created in the future), to i) publish, reproduce, distribute, display and store the Contribution, ii) translate the Contribution into other languages, create adaptations, reprints, include within collections and create summaries, extracts and/or, abstracts of the Contribution, iii) create any other derivative work(s) based on the Contribution, iv) to exploit all subsidiary rights in the Contribution, v) the inclusion of electronic links from the Contribution to third party material where-ever it may be located; and, vi) licence any third party to do any or all of the above.

For peer review only

All authors have completed the ICMJE uniform disclosure form at www.icmje.org/coi_disclosure.pdf and declare: no support from any organisation for the submitted work; no financial relationships with any organisations that might have an interest in the submitted work in the previous three years; no other relationships or activities that could appear to have influenced the submitted work.

**Conflicts of interest**: None

**Transparency:** The lead author (Michael Allen) confirms that the manuscript is an honest, accurate, and transparent account of the study being reported; that no important aspects of the study have been omitted.

**Funding**: This study was jointly funded by the National Institute of Health Research (NIHR) Collaboration for Leadership in Applied Health Research and Care for the South West Peninsula, and the South West Academic Health Science Network. The views and opinions expressed in this paper are those of the authors, and not necessarily those of the NHS, the National Institute for Health Research, or the Department of Health.

**Data sharing**: Data and code used in this study is available at https://github.com/MichaelAllen1966/1807_stroke_pathway

The repository includes code for pathway and machine learning models, and data used to train/test the machine learning model. It is provided under the MIT license, with no restrictions to use and no warranty.

**Ethical approval**: This study used anonymous secondary patient data only, collected as part of routine care audit. The study was conducted as part of a local service evaluation and improvement exercise sponsored by the South West Strategic Clinical Network. There was no access to patient identifiable information, no collecting of primary data, and no patient care was altered during the process of this study. There was no patient enrolment into the study. A decision that no formal ethical approval was made by the project steering group with representation from the South West Strategic Clinical Network, the National Institute of Health Research (NIHR) Collaboration for Leadership in Applied Health Research and Care for the South West Peninsula, and the South West Academic Health Science Network.

**Guarantor**: KS

**Contributorship**: MA, KP, MJ and KS conceived the study. RE reviewed and provided guidance on the machine learning aspects. TM and KP reviewed and provided guidance on the simulation aspects. AS conducted literature reviews and provided guidance on methodologies. MA wrote the algorithm code used in this study and performed the primary analysis. MJ and BB informed the base assumptions of the model, provided key literature and reviewed the output. MA produced the first draft of the paper, and all authors contributed significant editing to the paper. KS oversaw all stages of the project.

For peer review only

## Abstract

**Objectives:** To evaluate the application of clinical pathway simulation in machine learning, using clinical audit data, in order to identify key drivers for improving use and speed of thrombolysis at individual hospitals.

**Design:** Computer simulation modelling and machine learning.

**Setting:** Seven acute stroke units.

**Participants:** Anonymised clinical audit data for 7,864 patients.

**Results:** Three factors were pivotal in governing thrombolysis use: (1) the proportion of patients with a known stroke onset time (range 44% to 73%), (2) pathway speed (for patients arriving within 4 hours of onset: per-hospital median arrival-to-scan ranged from 11 to 56 minutes; median scan-to-thrombolysis ranged from 21 to 44 minutes) and (3) predisposition to use thrombolysis (thrombolysis use ranged from 31% to 52% for stroke patients scanned with 30 minutes left to administer thrombolysis). A pathway simulation model could predict the potential benefit of improving individual stages of the clinical pathway speed, whereas a machine learning model could predict the benefit of 'exporting' clinical decision making from one hospital to another, while allowing for differences in patient population between hospitals. By applying pathway simulation and machine learning together, we found a realistic ceiling of 15-25% use of thrombolysis across different hospitals and, in the seven hospitals studied, a realistic opportunity to double the number of patients with no significant disability that may be attributed to thrombolysis.

**Conclusions:** National clinical audit may be enhanced by a combination of pathway simulation and machine learning best allows for an understanding of key levers for improvement in hyper-acute stroke pathways, allowing for differences between local patient populations. These models, based on standard clinical audit data may be applied at scale while providing results at individual hospital level. The models facilitate understanding of variation, and levers for improvement, in stroke pathways, and help set realistic targets tailored to local populations.


## Strengths and limitations of this study

- The pathway simulation and methodology presented enhances the national clinical audit by providing insight into the inter-hospital variation in use and speed of thrombolysis.
- The methods allow reasonable thrombolysis targets to be set that are tailored to local stroke patient population characteristics.
- The methods (with code published in full) may be applied using Open Source Software, and may be applied at scale while providing tailored insight for individual hospitals.
- The methods are limited to information gathered in the national clinical audit for stroke, and do not include use of advanced imaging techniques for selection of patients for thrombolysis..

For peer review only

## Introduction

NHS England describe clinical audit as a way of identifying whether healthcare is being provided in accordance with agreed standards, and where improvements could be made to improve outcomes for patients [1]. Audits may be local or national. In England the National Quality Improvement Programmes (HQIP), on behalf of the NHS, is responsible for overseeing and commissioning more than 30 clinical audits which form the National Clinical Audit Programme [2]. These collect and analyse data supplied by local clinicians.

The National Audit covering stroke is the Sentinel Stroke National Audit Programme (SSNAP) [3] . Stroke is a leading cause of death and disability worldwide, with an estimated 5.9 million deaths and 33 million stroke survivors in 2010 [4]. In England, Wales and Northern Ireland 85,000 people are hospitalised with stroke each year [5], and stroke is ranked third as a cause of disability-adjusted life years in the UK over the last 25 years [6].

SSNAP collects longitudinal data on the processes and outcomes of stroke care up to six months post stroke for more than 90% of stroke admissions to acute hospitals in England, Wales and Northern Ireland. Every year data from approximately 85,000 patients is collected. SSNAP publishes quarterly and yearly analysis of results on its website [3].

SSNAP audit data is used for a wide range of research such as investigating how type of stroke effects clinical decisions [7], how socio-economic factors influence risk of stroke, care received, and outcomes [8] , and how care processes may vary by time of day and day of week [9].

In this paper we report on the potential of using simulation and machine learning to enhance the output of the SSNAP clinical audits. In particular we focus on the acute stroke pathway and clinical decision making leading to the use of thrombolysis for the treatment of acute stroke, the only licensed drug treatment for acute stroke, and one that is critically time-dependent [10] with little or no benefit after 4.5 hours from stroke onset. The population benefit from thrombolysis has been limited by slow uptake of the treatment, and in-hospital delays to the administration of thrombolysis [11–13].

In England, Wales and Northern Ireland 11.1% of confirmed acute stroke patients receive thrombolysis, but use in individual acutely admitting stroke teams varies from 0 to 24.5% [5]. The lowest 10% of acutely admitting stroke teams administer thrombolysis to fewer than 5.9% of patients, whereas the top 10% administer thrombolysis to more than 16.7%. Time from arrival to thrombolysis (‘door-to-needle’) also varies significantly. The fastest 10% of hospitals have door-to-needle times of 40 minutes or less, whereas the slowest 10% have door-to-needle times of 85 minutes or more [5]. There is therefore considerable variation between hospitals in the use, and speed, of thrombolysis for acute stroke patients.

The model described here has three components: 1) a clinical pathway model, 2) a clinical outcome model based on the speed and number of patients treated with thrombolysis, and 3) a clinical decision-making model based on machine learning.

Analysis of patient pathway data coupled with pathway modelling has previously allowed investigation and improvement of thrombolysis use in individual hospitals - increasing both the number of patients treated and reducing door-to-needle times [14,15]. These models have usually focused on the speed of the acute stroke pathway from arrival at hospital to giving thrombolysis, and have been tailored to use data available at an individual hospital [14].

For peer review only

For peer review only - http://bmjopen.bmj.com/site/about/guidelines.xhtml

Pathway modelling based on simulating process steps allows for good simulation of the speed of the stroke pathway, but cannot easily model differences in clinical decision making. We were interested in testing whether a model could dissect out the variation in thrombolysis rate that is dependent upon differences in patient populations (e.g. age or stroke severity) in different hospitals, from the differences that are dependent on the culture of decision making at different hospitals (e.g. more cautious vs more aggressive clinical decision making). A variety of machine learning techniques now exist [16], which are able to make good predictions on pre-existing multidimensional data over a binary or categorical outcome variable (such as whether a patient receives thrombolysis or not). These have the potential to add modelling of clinical decision making to a model of the acute stroke pathway, with the aim of predicting what decision (to thrombolyse or not) would be made for the same patient in different hospitals. Models may also be trained on a reference standard set of hospitals (regarded as centres of clinical excellence) and use of thrombolysis for any patient predicted using that 'benchmark clinical decision-making model'.

We have chosen to combine these three components of pathway, clinical outcome, and clinical decision-making modelling as that provides a more powerful and informative model than can be achieved by any single technique.

The aim of our work was to extend previous work on stroke thrombolysis pathway simulation in three significant ways: 1) to create a generic stroke thrombolysis pathway simulation model that could be easily applied to all hospitals in SSNAP, 2) extend the analysis to include factors other than door-to-needle times, with special focus on differences in clinical decision making as analysed and modelled with machine learning techniques, and 3) develop modelling framework that is open source and fast enough to run routine analysis at national level.

## Data

Anonymous SSNAP (Sentinel Stroke National Audit Programme) data was obtained from 7 hospitals in England, for patients with a confirmed stroke over a period of two years (2013-2014) for each hospital. These data were secondary data, collected during routine care. No patient identifiable information was obtained.

For the pathway simulation model, the dataset contained 7,871 patient records with complete data for 12 parameters regarding their characteristics and time stamped pathway location. These data represent out-of-hospital onset of stroke (which account for 94% of all admissions recorded in the SSNAP data used).

For machine learning, only those patients with a completed NIH Stroke Scale and who had at least 30 minutes left to give thrombolysis were used (1,862 patients). As a precaution to maintain complete patient anonymity 17 patients aged under 40 had their age censored to 40, and 6 patients over the age of 100 had their age censored to 100.

## Pathway simulation model

The pathway simulation model (shown schematically in figure 1) simulates the flow of patients through the acute stroke pathway to the point of thrombolysis. Model parameters were set for each hospital by sampling from distributions derived from anonymous data retrieved from SSNAP for each hospital. The model parameters, distribution types, and the values used are given in the online supplement.

For peer review only - http://bmjopen.bmj.com/site/about/guidelines.xhtml

For a patient to receive thrombolysis they must meet the following criteria: 1) stroke onset time known, 2) arrival at hospital within 4 hours of stroke onset, 3) have an ischaemic stroke and judged to be eligible for thrombolysis, and 4) be within the allowed thrombolysis time window (4.5 hours and 3 hours onset-to-treatment time for patients aged under 80, and 80+, respectively), when summing the process step times in the model. If a patient receives thrombolysis in the model then the probability of an additional good outcome (Modified Rankin Scale 0-1, no significant disability and able to carry out all usual activities) due to use of thrombolysis is calculated from the onset-to-treatment time and is based on the meta-analysis by Emberson et al. [10]

The pathway simulation model was validated by 1) random bootstrap sampling of 100 groups of 600 patients with varying overall thrombolysis use, comparing actual to predicted thrombolysis use, and 2) comparing actual and predicted thrombolysis use and speed across the seven hospitals.

## Clinical decision model (machine learning)

The clinical decision model aims to replicate the decision to give, or not give, treatment with thrombolysis for any given patient at any given hospital.

The model predicts whether an individual patient would receive thrombolysis or not from a set of 50 parameters defining the patient's characteristics, clinical well-being, and hospital attended. Following a comparison of methods, a Random Forests method was chosen (see online supplement for a list of all features used, and a comparison of different machine-learning models).

This model is intended to make decisions based only on clinical presentation, assuming that there is time to give thrombolysis. Patients were included if they had been scanned with 30 minutes left to give thrombolysis (allowing 4.5 hours and 3 hours from onset to treatment for patients aged under 80, and 80+, respectively).

The machine learning model was validated using stratified ten-fold validation, where the data is split into 10 subsets, and the model run 10 times (with each model run using 9 subsets for training and 1 subset held back for testing, with all data present in a test subset once and only once). All machine learning methods and validation coded in Python using the SciKit Learn machine learning library machine learning library [17].

## Patient and Public Involvement

Through this study we have used a panel of 4-5 stroke survivors or carers of stroke survivors. These were recruited through the NIHR PenCLAHRC Patient and Public Involvement group. These have met three times during the course of this project to help review aims, results and future plans.

# Results

## Pathway simulation

The pathway simulation model was validated by 1) random bootstrap sampling varying overall thrombolysis use, comparing actual to predicted thrombolysis use, and 2) comparing actual and predicted thrombolysis use and speed across the seven hospitals.

In the random sampling (figure 2), 600 patients (typical of an acute stroke unit) were sampled randomly, with resampling, from either the thrombolysis group or the no-thrombolysis group, to give varying samples with an overall thrombolysis use rate of 1-25%. 100 model runs were

For peer review only

performed. The model showed very good correlation (R-square 0.99) between actual and predicted values though the model slightly under-predicted actual thrombolysis use with predicted thrombolysis being, on average, 89% that of actual thrombolysis use.

The model was further validated by comparing modelled (predicted) use of thrombolysis with actual use of thrombolysis (table 1). Actual use of thrombolysis was based on modelling a one year period, with replicates of 100 runs (each with different random number seeds) in order to determine expected year-to-year variation. The model showed very good correlation (R-square 0.96) between actual and predicted values though the model again slightly under-predicted actual thrombolysis use with predicted thrombolysis being, on average, 90% that of actual thrombolysis use.

The difference between predicted and actual thrombolysis use is largely explained by the observation that 8% of thrombolysis in the SSNAP data set was given outside of the assumed allowable times for thrombolysis in the model; the model applies a stricter time cut-off than clinicians allow in reality.

The model was run with various ‘what-if?’ scenarios for each of the seven hospitals (table 2):

1. Base case: Model based on parameters derived from current hospital-specific performance

Scenario A: Door-to-needle time fixed at 30 minutes (by fixing arrival-to-scan time and scan-to-thrombolysis both at 15 minutes (with no variation in either time)

Scenario B: 60% of ischaemic stroke patients scanned with 30 minutes left to treat receive thrombolysis (An analysis of ECASS-3/IST-3 results concluded that 591 out of 992, or 60%, of ischaemic stroke patients arriving within 4 hours of stroke onset were suitable for thrombolysis [18]).

Scenario C: Onset time known fixed at 77% (national SSNAP upper quartile for year 2015/16 [5])

Combination of above

In order to achieve the greatest improvement in thrombolysis use in each of the seven hospitals, for three hospitals (hospitals 1, 2 and 6) it would be best to improve the speed of the pathway, for two hospitals (hospitals 4 and 5) it would be best to improve determination of stroke onset time, and for two (hospitals 3 and 7) it would be best to judge more patients as eligible for thrombolysis for those scanned with time left to treat. If a priority is to maximise clinical outcome then for five hospitals (hospitals 1, 2, 3, 5 and 6) it would be best to improve the speed of the pathway, for one hospital (hospital 4) it would be best to improve the determination of stroke onset time and for one (hospital 7) it would be best to judge more patients as eligible for thrombolysis for those scanned with time left to treat. Combining all changes in the model could produce thrombolysis rates up to 22-26%, and 23-26 additional non-disabled outcomes per 1,000 stroke patients admitted.

In the case of hospital 5, arrival-to-scan times could be *slowed* by an average of 30 minutes and clinical outcomes would still be greater if that hospital achieved a proportion of known stroke onset time equal to the national average.

## Clinical decision (machine learning) model

The Random Forest mode chosen has an 82% accuracy in predicting whether a patient received thrombolysis or not (see online supplement for more details on validation). A machine learning model may be trained on a subset of patients to investigate how the difference in thrombolysis use

For peer review only

between hospitals may be proportionally attributed to either the hospital or the local patient population. Table 3 shows the predicted use of thrombolysis in a set of patients that attend one hospital, based on decisions made from training at another hospital. Taking hospital 7 as an example, between 31% to 45% of the patients that currently attend hospital 7 (with time left to receive thrombolysis) might receive thrombolysis depending on which hospital decision making is used to train the model. Patient cohort also effects the predicted thrombolysis uses. Taking hospital 7 as an example again, if the model is trained on decisions made for patients attending hospital 7, and different hospital patient groups are then analysed in the model then thrombolysis use is predicted to be between 23% and 50% depending on the admitting hospital patient group analysed.

## Combining pathway simulation and machine learning

The output from machine learning may be incorporated into the stroke pathway model by using the machine learning model to make the decision in the pathway model about whether a patient is 'judged to be eligible for thrombolysis (for patients scanned with 30 minutes left to administer thrombolysis)'. This should tailor the clinical decision to the local population, without being affected by any particular hospital's predisposition to use thrombolysis. The 'judged to be eligible for thrombolysis' parameter in the pathway model may take its value from a machine learning model trained using a reference set of hospitals. The clinical decision making from these reference hospitals may be used to predict which of the patients from the hospital under study are eligible for thrombolysis.

Table 4 compares base case hospital performance (predicted thrombolysis use and clinical benefit) with the performance obtainable by a new realistic 'alternative' practice which is in part informed by the Random Forest machine learning model: (1) the proportion of patients with a known stroke onset time is set at the national median (67%) unless a hospital is already higher, (2) the door-to-needle time is set to 40 minutes for 90% of patients (20 minutes arrival-to-scan, and 20 minutes scan-to-needle) with the other 10% of patients not receiving a scan within 4 hours of arrival, and (3) the clinical decision to administer thrombolysis for those patients scanned with 30 minutes left to treat is set by the machine learning model trained from a reference hospital (this example uses the hospital that has the highest use of thrombolysis for those patients scanned with time to treat). Resulting thrombolysis targets vary from 16-25% depending on the hospital (base case 6 to 13%).

## Discussion

While there is no agreed benchmark for the proportion of stroke patients who should receive thrombolysis, it has been suggested that about half of stroke patients, if they arrive at hospital in time, could be clinically suitable for thrombolysis [18]. In practice the greatest proportion of patients treated with thrombolysis in English hyper-acute stroke services is close to 20% [5]. These figures contrast with an average 12% across England and Wales [5], and a range of 6%-14% across the seven acute stroke centres in our study. Internationally thrombolysis rates also appear on average to be lower than best-practice centres: for example rates have been reported to have been 14.6% in the Netherlands in 2012 [19] and 5.4% in USA in 2010 [20] (for patients aged 65 years or more).

Our pathway simulation model slightly under-predicted actual use of thrombolysis. This was mostly due to the model applying a strict cut-off of allowable time from onset of stroke to giving thrombolysis. In a real clinical setting a little bit of flexibility may be applied.

For peer review only

As patients move through the stroke pathway, an unknown stroke onset time would be the first key barrier to thrombolysis in our model – a barrier to thrombolysis previously noted [21]. Though a qualitative analysis of methods for determining the onset time of a stroke was beyond the scope of this study, it was clear that hospitals differed in how this information was gathered, such as whether they relied on information from paramedics only, or whether the hospital clinician would also investigate an unknown stroke onset time. In our modelling study we looked at the potential impact of reaching the national upper quartile for ascertaining stroke onset times. In one of the seven hospitals this factor was the single largest in attaining improvements to the thrombolysis rate. For patients with unknown time of onset, it may be possible to use advanced scanning methods to estimate stroke onset times and suitability for treatment [21,22] and further increase the population eligible for thrombolysis treatment. As advanced imaging techniques become established, the model could/should be extended to include this alternative pathway which our model does not currently include.

Reductions to in-hospital treatment delays have been the focus of previous modelling approaches [14,15], and the 'need for speed' has frequently been stressed [11,12]. Our study found that compared with current state it was reasonable to expect that improvements to pathway speed and reliability could achieve close to 50% increase in the number of disability free patients. Our modelling was based on consistently achieving 30 minutes door-to-needle times. It may be possible to be even more aggressive on process time as speeds of 20 minutes door-to-needle times have been reported [23]. Those hospitals in our study where paramedics took FAST-positive patients straight to the scanner (bypassing ED) had significantly faster arrival-to-scan times, and this may be a more widely applicable approach, although it was not responsible on its own for a higher thrombolysis rate in those centres, illustrating the multi-factorial influences on both thrombolysis rate and door-to-needle time; it may sometimes be best to accept slower pathway speeds in order to improve other factors in the pathway (such as ascertaining stroke onset time). A challenge for smaller hospitals is to have consistently fast processes 24 hours per day seven days per week; scanners must be rapidly available for stroke patients at any time of day and day of week.

The final hurdle investigated was in the confidence in using thrombolysis for patients scanned in time. Use of thrombolysis, when time allowed, varied significantly between hospitals. Physician uncertainty, or lack of confidence in thrombolysis, has previously been identified as a barrier to use of thrombolysis [24]. Despite meta-analysis demonstrating the clinical benefit of thrombolysis [10], the use and benefit of thrombolysis has still recently been under scrutiny [25]. This could certainly explain the large between-hospital variation in thrombolysis rates we observed for those patients scanned with time left to receive thrombolysis.

Scenarios modelled using our stand-alone pathway model used a standard 'optimum' rate for the clinical decision to use thrombolysis based on an analysis of the IST-3 trial. However this scenario assumes all hospitals receive patients who have similar characteristics overall to those in the IST-3 trial, and assumes that the IST-3 trial criteria should be a benchmark for all hospitals. The machine learning model allows for differences in patient populations between hospitals (for example reflecting different age demographics between different regions), and allows for differences in real-life (rather than clinical trial) decision making. Using results from the machine learning model, which focuses solely on clinical suitability for thrombolysis when there is time left to treat after the scan, enabled the pathway model to be refined and tailored further to local hospital populations. Machine learning models had an accuracy of 80-82% in predicting decisions made. The current

For peer review only

performance, while falling short of performance necessary for real-world decision making or guidance, is still useful in audit to understand variation in use of thrombolysis,

Using machine learning algorithms we found that differences in thrombolysis rate, for patients with time left to thrombolyse after the scan, can be explained by differences in patient population and in differences in decision making between hospitals. Rather than being based on a set of simple rules, our machine learning model learn from the clinical decision in real-life settings. A machine learning model allows prediction of potential thrombolysis use given the characteristics of the local population, but based on real-life clinical decision making at a range of hospitals, identifying those hospitals which appear to be outliers in their decision making. Bembernek et al. [18] have estimated that if the inclusion criteria of the IST-3 trial are used to determine suitable for thrombolysis, about 60% of ischaemic stroke patients (equivalent to 50% all stroke patients, if 85% of patients have ischaemic stroke could be suitable for thrombolytic treatment) if they arrive in time and the pathway is efficient. This overall figure however does not allow for differences in patient mix in different geographic areas. A clinician reviewing their own use of thrombolysis may conclude that their use is different because their patients are different. Machine learning confirms differences in the patients populations, but also suggests differences in clinical decision making. By applying machine learning, feedback may be given regarding their use of thrombolysis compared with other hospitals allowing for differences in local populations. This may be especially useful if a benchmark group of hospitals (acknowledged centres of excellence in stroke care) is used the train the model.

When applying 'stretch targets' (e.g. basing overall use of thrombolysis on an analysis of the IST-3 trial) to stroke pathways in the pathway model we found that maximum thrombolysis use could be about 22-26% of all emergency confirmed stroke admissions. This could have the benefit of producing another 22-27 people with no significant disability for every 1,000 confirmed stroke patients admitted, with potential to also improve outcomes for those who cannot be classified as having no significant disability even after use of thrombolysis. Using slightly less challenging targets for the acute stroke pathway process, and using machine learning to mimic decisions to thrombolyse adjusted for local populations we found that individual hospital targets could realistically be set in the 16-25% range, which would lead to another 16-25 people with no significant disability for every 1,000 patients admitted. The upper limit varied between hospitals based on the patient mix attending that hospital. Such results warn against dangers of setting any universal expectation of use of thrombolysis

This approach has the potential to be applied locally or, as it is based solely on SSNAP data, as part of the national audit of stroke services. The methods have the potential to be automated, allowing for incorporation into the quarterly stroke service audit conducted by SSNAP. Where clinical decision making appears to be significantly different from a reference group of hospital the machine learning model may be used to identify a small group of patients for review, where clinical decision making appears to be different from a model trained on an agreed benchmark set of hospitals.

Our work is not the first work to apply simulation or machine learning in stroke. Previous work has been published on using pathway simulation at an individual hospital level [14,15]. We build on previous work by using standardised data and an open simulation framework to enable pathway simulation to be performed at scale across all hospital covered by the national stroke audit. Previous work has also been published using machine learning to predict risk of stroke [26], and likely outcome [27–29]. Where our work adds novelty is firstly in the combination with pathway

For peer review only

simulation, and the use of machine learning to investigate differences in clinical decision making between hospitals, and to apply “what if?” scenario testing of what might the likely clinical benefit be of standardising clinical decision making in accordance with recognised centres of excellence, taking into account differences in local patient populations.

*Strengths and weakness*

A key strength of our study is that, since the models are based solely on SSNAP data, the models developed may be applied to all UK hospitals. The use of Open Source software and the provision of our code in a public repository should also facilitate easier adoption. Monks et al. [30]have noted that when modeling stroke pathways the level of detail of the model should depend on the questions being addressed. In our approach we consciously limit ourselves to the detail found in SSNAP data. A weakness, therefore, is that the models as they are presented cannot address questions that require data outside of the SSNAP data set. Our models highlight the improvements that can be achieved by an overall pathway stage (such as arrival-to-scan) but do not inform how improvement in that step could/should be achieved.

A strength of our modelling compared with normal approaches to stroke pathway modelling [30] is that we have used machine learning in order to both allow for differences in local stroke patient populations (e.g. differences in age, gender, and severity), and to understand differences in decision making between hospitals. A limitation of this approach is that the modelling is limited to hospital level, and will not uncover differences in use of thrombolysis by different practitioners within the same hospital. Nevertheless, the model will pick up overall organisational cultural attitudes towards use of thrombolysis in stroke (such as whether one hospital is more or less likely to use thrombolysis for patients with less severe stroke).

The use of Open Source software and the provision of our code in a public repository should also facilitate easier adoption, or refinement of the approach by others.

A weakness of this study is that it was limited in scope to seven hospitals. This methodology should preferably be validated on a larger data set (e.g. the national SSNAP data set).

Our model is deliberately limited to the SSNAP data set, as that is universally available across all stroke units in England and Wales. There are likely useful clinical features missing from the SSNAP data set (e.g. information from advanced imaging). As the SSNAP data set grows we would expect accuracy of the machine learning model to increase beyond the current 80%, but we believe the model is already sufficiently useful to add more insight to the routine SSNAP audit.

In this model we have considered only use of thrombolysis. As the SSNAP data set grows we would expect a similar approach to be useful in identifying levels to increase use and speed of thrombectomy (by introducing inter-hospital transfer, when required, into the clinical pathway model, and also building a learning model on use of thrombectomy to identify differences in selection of patients for thrombectomy). Additionally, we have focussed on the most usual path to thrombolysis – those arriving and scanned with time left to treat within the normal thrombolysis time limits. Advanced imaging may be used to select further patients [31]; as advanced imaging becomes more commonplace then the model would be best extended to include this additional pathway.

In this study we have not focussed on how implementation of changes may be brought about in different hospitals. It is possible that some improvements in the pathway (such as rapid availability

For peer review only

to scan 24/7) may only be possible by centralisation of services into larger units who may have more resources to deploy [32–34]. Our model will show the benefit of reducing pathway speed and variability, but does not imply such a change is necessary always possible in smaller hospitals.

## Conclusions

The stroke clinical audit reports on thrombolysis usage (percentage of patients receiving thrombolysis), and time to thrombolysis. Inter-hospital variation in use of thrombolysis may be due to (1) differences in stroke pathway, (2) differences in patient population characteristics, or (3) differences in clinical decision making. In our study we present a method that qualitatively analyses key components of the stroke pathway in a manner that may be applied at scale while producing individualised results for each hospital, highlighting how thrombolysis use and speed may best be improved. We believe this type of approach will augment current outputs from national clinical audits.

For peer review only

## References


1 NHS England. Clinical Audit. 2018.https://www.england.nhs.uk/clinaudit/ (accessed 5 Oct 2018).

2 National quality improvement programmes (HQIP). The National Clinical Audit Programme. https://www.hqip.org.uk/a-z-of-nca/ (accessed 5 Oct 2018).

3 Royal College Of Physians. Sentinel Stroke National Audit Programme (SSNAP): Web Site. https://www.strokeaudit.org/

4 Feigin VL, Forouzanfar MH, Krishnamurthi R, *et al.* Global and regional burden of stroke during 1990-2010: Findings from the Global Burden of Disease Study 2010. *The Lancet* 2014;**383**:245–255. doi:10.1016/S0140-6736(13)61953-4

5 Sentinel Stroke Audit Programme (SSNAP). *Apr2015Mar2016-AnnualResultsPortfolio*. 2016.

6 Newton JN, Briggs ADM, Murray CJL, *et al.* Changes in health in England, with analysis by English regions and areas of deprivation, 1990-2013: A systematic analysis for the Global Burden of Disease Study 2013. *The Lancet* 2015;**386**:2257–2274. doi:10.1016/S0140-6736(15)00195-6

7 Parry-Jones AR, Paley L, Bray BD, *et al.* Care-limiting decisions in acute stroke and association with survival: analyses of UK national quality register data. *Int J Stroke* 2016;**11**:321–31. doi:10.1177/1747493015620806

8 Bray BD, Paley L, Hoffman A, *et al.* Socioeconomic disparities in first stroke incidence, quality of care, and survival: a nationwide registry-based cohort study of 44 million adults in England. *Lancet Public Health* 2018;**3**:e185–93. doi:10.1016/S2468-2667(18)30030-6

9 Bray BD, Cloud GC, James MA, *et al.* Weekly variation in health-care quality by day and time of admission: a nationwide, registry-based, prospective cohort study of acute stroke care. *The Lancet* 2016;**388**:170–7. doi:10.1016/S0140-6736(16)30443-3

10 Emberson J, Lees KR, Lyden P, *et al.* Effect of treatment delay, age, and stroke severity on the effects of intravenous thrombolysis with alteplase for acute ischaemic stroke: A meta-analysis of individual patient data from randomised trials. *The Lancet* 2014;**384**:1929–1935. doi:10.1016/S0140-6736(14)60584-5

11 Meretoja A, Keshtkaran M, Saver JL, *et al.* Stroke thrombolysis: Save a minute, save a day. *Stroke* 2014;**45**:1053–1058. doi:10.1161/STROKEAHA.113.002910

12 Saver JL. Time is brain - Quantified. *Stroke* 2006;**37**:263–266. doi:10.1161/01.STR.0000196957.55928.ab

13 Lyden P. Thrombolytic Therapy for Acute Stroke — Not a Moment to Lose. *N Engl J Med* 2008;**359**:1393–1395. doi:10.1056/NEJMe0806335

14 Monks T, Pitt M, Stein K, *et al.* Maximizing the population benefit from thrombolysis in acute ischemic stroke: A modeling study of in-hospital delays. *Stroke* 2012;**43**:2706–2711. doi:10.1161/STROKEAHA.112.663187


For peer review only

15 Lahr M, van der Zee D-J, Luijckx G, *et al.* A simulation-based approach for improving utilization of thrombolysis in acute brain infarction. *Med Care* 2013;**51**:1101–1105. doi:10.1097/MLR.0b013e3182a3e505

16 Kotsiantis SB, Zaharakis I, Pintelas P. Supervised machine learning: A review of classification techniques. *Informatica* 2007;**31**:249–268. doi:10.1115/1.1559160

17 Pedregosa F, Varoquaux G, Gramfort A, *et al.* Scikit-learn: Machine Learning in Python. *J Mach Learn Res* 2011;**12**:2825–30.

18 Bembenek J, Kobayashi A, Sandercock P, *et al.* How many patients might receive thrombolytic therapy in the light of the ECASS-3 and IST-3 data? *Int J Stroke* 2010;**5**:430–1. doi:10.1111/j.1747-4949.2010.00479.x

19 Scherf S, Limburg M, Wimmers R, *et al.* Increase in national intravenous thrombolysis rates for ischaemic stroke between 2005 and 2012: is bigger better? *BMC Neurol* 2016;**16**:53. doi:10.1186/s12883-016-0574-7

20 George BP, Asemota AO, Dorsey ER, *et al.* United States trends in thrombolysis for older adults with acute ischemic stroke. *Clin Neurol Neurosurg* 2015;**139**:16–23. doi:10.1016/j.clineuro.2015.08.031

21 Aoki J, Kimura K, Iguchi Y, *et al.* FLAIR can estimate the onset time in acute ischemic stroke patients. *J Neurol Sci* 2010;**293**:39–44. doi:10.1016/j.jns.2010.03.011

22 Aoki J, Kimura K, Iguchi Y, *et al.* FLAIR can estimate the onset time in acute ischemic stroke patients. *J Neurol Sci* 2010;**293**:39–44. doi:10.1016/j.jns.2010.03.011

23 Meretoja A, Strbian D, Mustanoja S, *et al.* Reducing in-hospital delay to 20 minutes in stroke thrombolysis. *Neurology* 2012;**79**:306–313. doi:10.1212/WNL.0b013e31825d6011

24 Kwan J, Hand P, Sandercock P. A systematic review of barries to delivery of thrombolysis for acute stroke. *Age Ageing* 2004;**33**:116–121. doi:10.1093/ageing/afh064

25 Alper BS, Malone-Moses M, McLellan JS, *et al.* Thrombolysis in acute ischaemic stroke: time for a rethink? *Bmj* 2015;**350**:h1075–h1075. doi:10.1136/bmj.h1075

26 Khosla A, Cao Y, Lin CC-Y, *et al.* An integrated machine learning approach to stroke prediction. *Proc 16th ACM SIGKDD Int Conf Knowl Discov Data Min - KDD 10* 2010;:183. doi:10.1145/1835804.1835830

27 Rehme AK, Volz LJ, Feis D-L, *et al.* Identifying Neuroimaging Markers of Motor Disability in Acute Stroke by Machine Learning Techniques. *Cereb Cortex* 2015;**25**:3046–56. doi:10.1093/cercor/bhu100

28 Asadi H, Dowling R, Yan B, *et al.* Machine learning for outcome prediction of acute ischemic stroke post intra-arterial therapy. *PLoS ONE* 2014;**9**:14–19. doi:10.1371/journal.pone.0088225

29 Bentley P, Ganesalingam J, Carlton Jones AL, *et al.* Prediction of stroke thrombolysis outcome using CT brain machine learning. *NeuroImage Clin* 2014;**4**:635–40. doi:10.1016/j.nicl.2014.02.003

For peer review only

30 Monks T, van der Zee D-J, Lahr M, *et al.* A framework to accelerate simulation studies of hyperacute stroke systems. *Oper Res Health Care* 2017;**15**:57–67. doi:10.1016/j.orhc.2017.09.002

31 MRI-Guided Thrombolysis for Stroke with Unknown Time of Onset | NEJM. https://www.nejm.org/doi/full/10.1056/NEJMoa1804355 (accessed 18 Jul 2019).

32 Lahr MMH, van der Zee D-J, Luijckx G-J, *et al.* Centralising and optimising decentralised stroke care systems: a simulation study on short-term costs and effects. *BMC Med Res Methodol* 2017;**17**:5. doi:10.1186/s12874-016-0275-3

33 Morris S, Hunter RM, Ramsay AIG, *et al.* Impact of centralising acute stroke services in English metropolitan areas on mortality and length of hospital stay: difference-in-differences analysis. *Bmj* 2014;**349**:g4757–g4757. doi:10.1136/bmj.g4757

34 Ramsay AIG, Morris S, Hoffman A, *et al.* Effects of centralizing acute stroke services on stroke care provision in two large metropolitan areas in England. *Stroke* 2015;**46**:2244–2251. doi:10.1161/STROKEAHA.115.009723

For peer review only

## Figure legends

*Figure 1. Modelled sequence of steps of the emergency stroke pathway leading to thrombolysis.*

*Figure 2. Validation of the pathway simulation model, comparing actual to modelled (predicted) thrombolysis based on random sampling of all data. Samples were 600 points (representing typical acute stroke unit admission numbers) chosen randomly with resampling from patients given or not given thrombolysis to create a range of thrombolysis use examples. Points show mean predicted thrombolysis use from 100 runs, with each run modelling one year.*

For peer review only

*Table 1. Comparison of actual vs. modelled hospital performance.*

| *Hospital* | *1* | *2* | *3* | *4* | *5* | *6* | *7* |
|---|---|---|---|---|---|---|---|
| *Actual thrombolysis (%)* | *13.7* | *8.0* | *8.4* | *7.0* | *8.5* | *14.5* | *9.2* |
| *Model thrombolysis (%)* | *12.9* | *7.1* | *7.6* | *6.3* | *8.1* | *12.0* | *8.0* |
| *Actual onset to thrombolysis (hrs)* | *2.4* | *2.6* | *2.9* | *2.5* | *2.3* | *2.5* | *2.5* |
| *Model onset to thrombolysis (hrs)* | *2.6* | *2.6* | *3.0* | *2.6* | *2.7* | *2.6* | *2.5* |

For peer review only

*Table 2: Predicted thrombolysis use and clinical benefit across all modelled hospitals (1 to 7). Data shows: (Base) Model based on parameters derived from current performance; (A) Arrival-to-scan and scan-to-thrombolysis both fixed at 15 min (with no variation in either time); (B) Judged to be eligible for thrombolysis fixed at 60%; (C) Onset time known fixed at 77%; Combinations of the above. Results show mean and ±95% confidence limits (100 runs).*

| | Hospital | Base | A | B | C | ABC |
|---|---|---|---|---|---|---|
| Thrombolysis use (%) | 1 | 12.7 (0.3) | 17.6 (0.3) | 13.9 (0.3) | 14.6 (0.3) | 21.5 (0.3) |
| | 2 | 7.0 (0.2) | 10.4 (0.3) | 10.8 (0.2) | 10.0 (0.3) | 23.4 (0.3) |
| | 3 | 7.7 (0.2) | 9.7 (0.2) | 11.9 (0.2) | 10.9 (0.2) | 21.7 (0.3) |
| | 4 | 6.2 (0.2) | 6.8 (0.2) | 11.2 (0.2) | 11.3 (0.3) | 20.7 (0.3) |
| | 5 | 7.9 (0.3) | 10.4 (0.3) | 10.0 (0.3) | 13.2 (0.4) | 22.1 (0.5) |
| | 6 | 12.3 (0.3) | 16.3 (0.4) | 14.5 (0.4) | 12.7 (0.3) | 20.2 (0.4) |
| | 7 | 8.0 (0.2) | 9.4 (0.2) | 14.0 (0.3) | 10.8 (0.2) | 22.5 (0.3) |
| Additional good outcomes per 1,000 admissions | 1 | 11.1 (0.2) | 17.2 (0.3) | 12.2 (0.2) | 12.8 (0.3) | 21.0 (0.3) |
| | 2 | 6.1 (0.2) | 10.2 (0.3) | 9.3 (0.2) | 8.6 (0.2) | 23.0 (0.4) |
| | 3 | 6.5 (0.2) | 10.1 (0.2) | 10.0 (0.2) | 9.1 (0.2) | 22.5 (0.3) |
| | 4 | 5.4 (0.2) | 6.7 (0.2) | 9.8 (0.2) | 9.9 (0.2) | 20.0 (0.3) |
| | 5 | 7.0 (0.3) | 10.8 (0.4) | 8.7 (0.3) | 11.8 (0.4) | 23.1 (0.5) |
| | 6 | 10.6 (0.3) | 16.0 (0.4) | 12.5 (0.3) | 11.0 (0.3) | 19.9 (0.4) |
| | 7 | 7.1 (0.2) | 9.5 (0.3) | 12.4 (0.2) | 9.6 (0.2) | 22.9 (0.3) |

For peer review only

*Table 3. Actual and predicted thrombolysis use (for patients with patients scanned with time left to thrombolysis) if the decision to give thrombolysis is based on decisions made by a Random Forest model trained at different hospitals. The columns therefore represent the likely difference in thrombolysis use due to differences in decision making.*

*Actual thrombolysis use by hospital*

| 1 | 2 | 3 | 4 | 5 | 6 | 7 |
|---|---|---|---|---|---|---|
| 52 | 35 | 48 | 33 | 49 | 44 | 31 |

*Predicted thrombolysis use at each hospital depending on which hospital is used to train the decision model, and which hospital patients actually attend*

| | | Hospitals patients actually attend | | | | | | |
|---|---|---|---|---|---|---|---|---|
| | | 1 | 2 | 3 | 4 | 5 | 6 | 7 |
| Hospital used to train model | 1 | 52 | 42 | 58 | 50 | 67 | 57 | 45 |
| | 2 | 48 | 35 | 55 | 36 | 46 | 37 | 29 |
| | 3 | 53 | 38 | 48 | 46 | 58 | 41 | 34 |
| | 4 | 40 | 28 | 48 | 33 | 52 | 29 | 26 |
| | 5 | 50 | 36 | 50 | 40 | 49 | 45 | 37 |
| | 6 | 49 | 32 | 55 | 44 | 59 | 44 | 39 |
| | 7 | 42 | 23 | 42 | 31 | 50 | 36 | 31 |

For peer review only

*Table 4. Combining pathway simulation and machine learning. Predicted thrombolysis use and clinical benefit (additional good outcomes per 1,000 admitted patients) across all modelled hospitals (1 to 7) from the pathway simulation. Data shows: (Base) Model based on parameters derived from current performance; Alternative 'realistic target' settings, fixing the proportion of known stroke onset times to the national SSNAP average (67% median) unless the hospital currently performs higher, fixing arrival-to-scan and scan-to-needle to 20 minutes each (with 10% of patients not scanned within 4 hours), and fixing the proportion of treatable patients (scanned with 30 minutes left to treat) according to the output of the machine learning model based on the hospital with the maximum predicted proportion given thrombolysis. Data shows mean and 95% confidence intervals.*

| Hospital | Thrombolysis use (%) | | Additional good outcomes per 1,000 admissions | |
|---|---|---|---|---|
| | Current | Alternative | Current | Alternative |
| 1 | 12.9 (0.3) | 18.6 (0.3) | 11.3 (0.2) | 17.4 (0.3) |
| 2 | 7.1 (0.2) | 15.3 (0.3) | 6.1 (0.2) | 14.6 (0.3) |
| 3 | 7.6 (0.2) | 22.0 (0.3) | 6.3 (0.1) | 21.4 (0.3) |
| 4 | 6.3 (0.2) | 17.2 (0.3) | 5.5 (0.2) | 16.0 (0.3) |
| 5 | 8.1 (0.3) | 25.3 (0.5) | 7.1 (0.3) | 25.2 (0.5) |
| 6 | 12.0 (0.4) | 23.4 (0.4) | 10.5 (0.3) | 22.0 (0.4) |
| 7 | 8.0 (0.2) | 16.9 (0.3) | 7.1 (0.2) | 16.5 (0.3) |

For peer review only

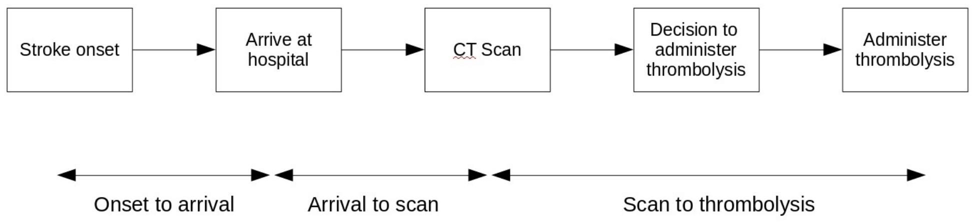


Figure 1. Modelled sequence of steps of the emergency stroke pathway leading to thrombolysis.

494x122mm (72 x 72 DPI)

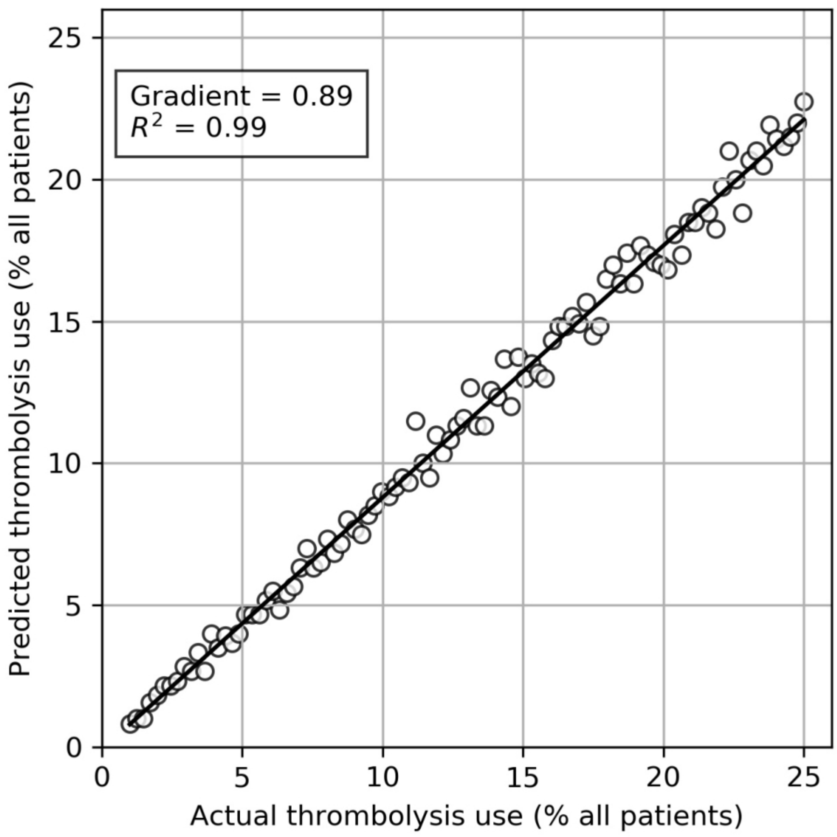


Figure 2. Validation of the pathway simulation model, comparing actual to modelled (predicted) thrombolysis based on random sampling of all data. Samples were 600 points (representing typical acute stroke unit admission numbers) chosen randomly with resampling from patients given or not given thrombolysis to create a range of thrombolysis use examples. Points show mean predicted thrombolysis use from 100 runs, with each run modelling one year.

127x127mm (300 x 300 DPI)

# Can clinical audit be enhanced by pathway simulation and machine learning? An example from the acute stroke pathway.

# Supplement

## 1 GitHub code repository

Both the pathway simulation and the machine learning models use Free Open Source Software (both models are based on Python). All code for the model (with anonymous patient data) is available at https://github.com/MichaelAllen1966/stroke_thrombolysis_pathway

## 2 Data

Anonymous SSNAP data was obtained from 7 hospitals in England, for patients with a confirmed stroke over a period of two years (2013-2014) for each hospital. These data were secondary data, collected during routine care. No patient identifiable information was obtained.

For the pathway simulation model, the dataset contained 7,871 patient records with complete data for 12 parameters regarding their characteristics and time stamped pathway location. These data represent out-of-hospital onset of stroke (which account for 94% of all admissions recorded in the SSNAP data used).

For machine learning, only those patients with a completed NIH Stroke Scale and who had at least 30 minutes left to give thrombolysis were used (1,862 patients). As a precaution to maintain complete patient anonymity 17 patients aged under 40 had their age censored to 40, and 6 patients over the age of 100 had their age censored to 100.

### 2.1 Patient characteristics

Table 1 shows the characteristic of patients used for modelling. The clinical pathway simulation model used all patients with out-of-hospital onset, whereas as the machine learning thrombolysis decision model was based on those patients scanned with 30 minutes left for treatment, and who had a NIHSS score.



For peer review only

*Table 1: Characteristics of patients used in the models*

| | Mean (+standard deviation where appropriate) | |
|---|---|---|
| | All out of hospital admissions | All patients with 30 minutes left to treat after scan (and NIHSS recorded) |
| Patients | 7871 | 1862 |
| Thrombolysis given | 0.09 | 0.40 |
| Male | 0.48 | 0.52 |
| Age | 76 (13) | 74 (13) |
| Age 80+ | 0.48 | 0.37 |
| Onset time known | 0.55 | 1.00 |
| Arrive by ambulance | 0.85 | 0.95 |
| Arrival within 4 hours of known onset | 0.38 | 1.00 |
| Age 80+ 2.5 hour arrival, others 4hr arrival | 0.35 | 1.00 |
| Scanned | 0.99 | 1.00 |
| # Comorbidities | 1.00 | 1.18 |
| 2+ comorbidities | 0.36 | 0.35 |
| Congestive HF | 0.05 | 0.05 |
| Hypertension | 0.48 | 0.47 |
| Atrial Fib | 0.21 | 0.22 |
| Diabetes | 0.17 | 0.16 |
| Previous TIA | 0.30 | 0.28 |
| Co-mordity | 0.72 | 0.71 |
| Antiplatelet | 0.38 | 0.08 |
| Anticoag before stroke | 0.50 | 0.10 |
| NIHSS recorded | 0.66 | 1.00 |
| NIHSS on Arrival | 8.0 (7.7) | 10.4 (7.8) |
| Onset to scan with 30 min left | 0.25 | 1.00 |
| Arrival to stroke unit (min) | 858 (2386) | 86 (38) |
| Arrival to scan | 375 (1389) | 29 (27) |
| Onset to scan | 969 (11119) | 116 (44) |
| Scan to thrombolysis | 42 (27) | 42 (27) |
| Arrival to thrombolysis | 69 (35) | 68 (34) |
| Onset to thrombolysis | 156 (69) | 148 (47) |



For peer review only - http://bmjopen.bmj.com/site/about/guidelines.xhtml

For peer review only

Table 2 shows key patients characteristics, summarised by hospital, for patients with 30 minutes left to treat after scan (and NIHSS recorded).

*Table 2: Key patients characteristics, summarised by hospital, for patients with 30 minutes left to treat after scan (and NIHSS recorded)*

| | Hospital | | | | | | |
|---|---|---|---|---|---|---|---|
| | 1 | 2 | 3 | 4 | 5 | 6 | 7 |
| Age | 74 (13) | 75 (14) | 72 (12) | 75 (12) | 75 (11) | 74 (13) | 75 (12) |
| Age 80+ | 0.38 | 0.42 | 0.25 | 0.41 | 0.38 | 0.34 | 0.39 |
| Male | 0.51 | 0.49 | 0.54 | 0.49 | 0.43 | 0.54 | 0.56 |
| NIHSS on Arrival | 11.8 (8.0) | 9.5 (8.1) | 10.6 (6.9) | 11.2 (8.3) | 12.2 (7.9) | 9.7 (6.8) | 9.0 (7.4) |

# 3 Pathway simulation model

## 3.1 High level model description

The simulation model mimics the flow of individual patients through the acute stroke pathway as shown in figure 1). The simulation model builds a 'virtual population' of patients which travel through the model. The characteristics of the patients are taken from sampling from distributions based on the patient-level SSNAP data for each individual hospital.

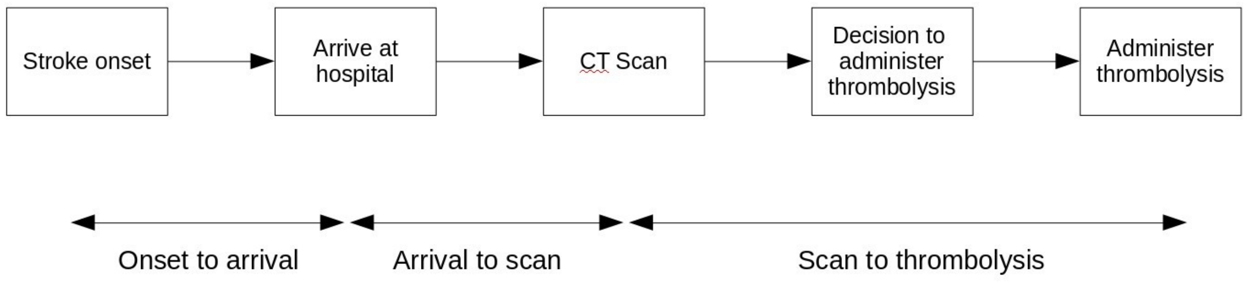


*Figure 1: High level view of patient flow through model*

For a patient to receive thrombolysis in the model the patient must:

1. Arrive within four hours of known onset
2. Receive a scan within 4 hours of arrival and receive a scan with at least 20 minutes left to treat with thrombolysis (onset-to-treatment time limits are 270 minutes for patients aged less than 80, and 180 minutes for patients aged 80+).
3. Have an ischaemic stroke and be considered eligible for thrombolysis



For peer review only - http://bmjopen.bmj.com/site/about/guidelines.xhtml

For patients receiving thrombolysis the probability of an additional good outcome is calculated according to the analysis of Emberson *et al.[1]*.

The model runs in Python (using NumPy arrays to represent a population of patients). Each run of the model mimics 100 independent one year of admissions to a given hospital.

## 3.2 Model outputs

For each scenario, the model reports for the following (from 100 independent years):

- Mean, standard deviation, 5th percentile, lower quartile, median, upper quartile, and 95th percentile of:
  - Baseline good outcomes (MRS0-1)
  - Thrombolysis use (% of all admissions)
  - Additional good outcomes per 1,000 admissions

## 3.3 Hospital performance parameters used in the pathway simulation model

The parameters used in the thrombolysis pathway simulation model are shown in table 3. All parameters are derived from data recorded in SSNAP.

*Table 3: Parameters used in the stroke pathway simulation model (patients with in-hospital stroke onset excluded)*

| Parameter | Distribution | Range used (base case) |
|---|---|---|
| Allowed onset-to-treatment time for age up to 80 (mins) | Fixed | 270 |
| Allowed onset-to-treatment time for age 80 +(mins) | Fixed | 180 |
| Age 80+ (patients arriving within 4 hours of known onset) | Bernoulli | 0.363-0.523 |
| Arrivals per year | fixed | 300-800 |
| Onset time known | Bernoulli | 0.432-0.738 |
| Onset-to-arrival <4hrs | Bernoulli | 0.677-0.706 |
| Onset-to-arrival time (mins) | Log (ln) normal | Mean (ln): 4.417-4.609<br>StdDev (ln): 0.480-0.573 |
| Arrival-to-scan <4hrs | Bernoulli | 0.783-0.979 |
| Arrival-to-scan time (mins) | Log (ln) normal | Mean (ln): 2.477-3.789<br>StdDev (ln): 0.748-1.271 |
| Ischaemic stroke | Bernoulli | 0.770-0.896 |
| Eligible for thrombolysis | Bernoulli | 0.342-0.577 |
| Scan-to-thrombolysis (mins) | Log (ln) normal | Mean (ln): 23.087-3.80<br>StdDev (ln): 0.560-0.863 |



For peer review only - http://bmjopen.bmj.com/site/about/guidelines.xhtml

*Notes:*

1. *The allowed onset-to-treatment time for patients aged up to 80 is 4.5 hours.*
2. *The allowed onset-to-treatment time for patients aged 80+ is 3 hours.*
3. *The proportion of patients aged 80+ varies between hospitals.*
4. *The number of arrivals is the number of confirmed stroke patients in SSNAP per year*
5. *'Onset time known' is binary yes/no. Known onset time may be recorded as precise or estimated in SSNAP. Those without a known onset time cannot be treated with thrombolysis in the model.*
6. *'Onset to arrival <4hrs' is the proportion of patients with known stroke onset that arrive within 4 hours of onset. Those arriving more than 4 hours after onset cannot be treated with thrombolysis in the model.*
7. *'Onset to arrival time' is a log-normal distribution. It is applied only to those patients arriving within 4 hours of known stroke onset.*
8. *'Arrival to scan time <4hrs' is the proportion of patients (those with known stroke onset time and arriving within 4 hours of onset) that receive a CT head scan within 4 hours of arrival.*
9. *'Arrival to scan time' is a log-normal distribution. It is applied only to those patients arriving within 4 hours of known stroke onset, and receiving a scan within 4 hours of arrival.*
10. *'Ischaemic stroke' is the proportion of patients with ischaemic (rather than haemorrhagic stroke). It is applied only to those patients arriving within 4 hours of known stroke onset, and receiving a scan within 4 hours of arrival.*
11. *'Eligible for thrombolysis' is the proportion of ischaemic stroke patients (arriving within 4 hours of known stroke onset, and scanned within 4 hours of arrival) who are considered clinically eligible for thrombolysis. This figure is obtained by examining the proportion of ischaemic stroke patients who are scanned with at least 30 minutes left to receive thrombolysis who are given thrombolysis.*
12. *'Scan to thrombolysis' is a log-normal distribution. It is applied only to those patients arriving within 4 hours of known stroke onset, receiving a scan within 4 hours of arrival, and are ischaemic strokes considered eligible for thrombolysis*

The value of parameters used for each hospital's base case is shown in table 4.



For peer review only - http://bmjopen.bmj.com/site/about/guidelines.xhtml

*Table 4: Model input parameters for each of the seven hospitals in the study*

| Hospital | 1 | 2 | 3 | 4 | 5 | 6 | 7 |
|---|---|---|---|---|---|---|---|
| arrivals per year | 600 | 600 | 800 | 700 | 300 | 350 | 700 |
| Age 80+ (for patients arriving within 4hrs of known onset) | 0.492 | 0.523 | 0.363 | 0.486 | 0.422 | 0.490 | 0.475 |
| onset known | 0.679 | 0.537 | 0.541 | 0.432 | 0.460 | 0.738 | 0.562 |
| known arrival within 4hrs | 0.684 | 0.698 | 0.695 | 0.677 | 0.697 | 0.706 | 0.704 |
| onset arrival mins μ | 4.568 | 4.471 | 4.543 | 4.609 | 4.417 | 4.539 | 4.445 |
| onset arrival mins σ | 0.513 | 0.539 | 0.480 | 0.519 | 0.550 | 0.505 | 0.573 |
| scan within 4 hrs | 0.844 | 0.783 | 0.940 | 0.979 | 0.848 | 0.845 | 0.910 |
| arrival scan arrival mins μ | 3.645 | 3.421 | 3.789 | 2.477 | 3.660 | 3.409 | 3.151 |
| arrival scan arrival mins σ | 0.905 | 1.097 | 0.748 | 1.271 | 0.812 | 0.980 | 1.012 |
| ischaemic stroke | 0.865 | 0.896 | 0.820 | 0.864 | 0.831 | 0.770 | 0.859 |
| ischaemic eligible | 0.557 | 0.386 | 0.383 | 0.344 | 0.469 | 0.511 | 0.342 |
| scan needle mins μ | 3.087 | 3.547 | 3.800 | 3.695 | 3.522 | 3.461 | 3.686 |
| scan needle mins σ | 0.863 | 0.732 | 0.583 | 0.560 | 0.608 | 0.732 | 0.587 |
| thrombolysis rate (actual) | 0.137 | 0.080 | 0.084 | 0.070 | 0.085 | 0.145 | 0.092 |

### 3.3.1 Correlation between input parameters (patient level)

Table 5 shows correlation between model input parameters. Of the key steps in the model (arrival to scan time, scan to thrombolysis, proportion of patients receiving thrombolysis if there is time to treat), the strongest correlation (R-square) is -0.16.

*Table 5: Correlation between model input parameters (for patients arriving at an acute stroke unit within 4 hours of stroke onset)*

| | A | B | C | D | E | F | G | H |
|---|---|---|---|---|---|---|---|---|
| A | NA | 0.03 | -0.04 | 0.04 | -0.30 | -0.02 | -0.12 | -0.04 |
| B | 0.03 | NA | -0.13 | 0.11 | -0.49 | -0.04 | -0.24 | -0.13 |
| C | -0.04 | -0.13 | NA | -0.65 | 0.51 | 0.00 | 0.21 | NA |
| D | 0.04 | 0.11 | -0.65 | NA | -0.36 | 0.01 | -0.15 | -0.16 |
| E | -0.30 | -0.49 | 0.51 | -0.36 | NA | 0.06 | 0.35 | NA |
| F | -0.02 | -0.04 | 0.00 | 0.01 | 0.06 | NA | NA | NA |
| G | -0.12 | -0.24 | 0.21 | -0.15 | 0.35 | NA | NA | NA |
| H | -0.04 | -0.13 | NA | -0.16 | NA | NA | NA | NA |

A: Age 80+
B: Onset to arrival (min)
C: Scanned within 4hrs arrival
D: Arrival to scan
E: Onset to scan with 30 min left



For peer review only - http://bmjopen.bmj.com/site/about/guidelines.xhtml

F: Scan to thrombolysis
G: Thrombolysis given
H: Thrombolysis given if 30 minutes left to treat

## 3.4 Model steps

The model loads input parameters for multiple scenarios. For each scenario the following steps are performed:

1. Create an array representing all patient arrivals for one year (note: in the following steps all values are assigned to all patients, though they are only applied as appropriate)
2. Assign patient as aged 80+ or not (distribution os for patients who arrive within four hours of known onset)
3. Assign allowed onset-to-needle (depending on age group)
4. Assign onset time known or not
5. Assign onset to-arrival within 4 hours of known stroke onset
6. Assign onset-to-arrival time
7. Assign arrival to scan within 4 hours or not
8. Assign arrival to scan times
9. Assign time left for thrombolysis decision
10. Assign whether patients has all of: onset time known, onset-to-arrival <4hrs, arrival to scan <4hrs, and time left to receive thrombolysis (20 minutes time window remaining)
11. Assign whether patient has ischaemic stroke or not
12. Assign whether patient is eligible for thrombolysis
13. Assign desire to treat (based on steps 10, 12 & 12 all being positive)
14. Assign scan to needle time
15. Check onset-to-needle time is within licence time (+15 minutes to allow for over-run in the decision-to-treat to treatment stage).
16. Assign thrombolysis given (based on steps 13 & 15 both being positive)
17. Set basline probability of good outcome (based on age group), and convert to odds.
18. Assign odds-ratio of good outcome based on giving thrombolysis at assigned onset-to-treatment tie
19. Adjust odds of good outcome based on assigned odds-ratio in step 18, Convert back to probability.
20. Calculate additional probability of good outcome if given thrombolysis
21. Sum additional probabilities of good outcomes for all those treated (step 16)



For peer review only - http://bmjopen.bmj.com/site/about/guidelines.xhtml

22. Save proportion of admissions given thrombolysis (step 16) and the number of additional good outcomes (step 21).

## 3.5 The relationship between time to treatment and outcome

The effectiveness of thrombolysis as a function of time to treatment is taken from Emberson et al. [1], and is shown in figure 2. The model uses odds-ratio of a good outcome (Modified Rankin Scale 0-1). The baseline probabilities of a good outcome for patients aged less than 80, and 80+ are 0.350 and 0.132 respectively (equivalent odds = 0.538 and 0.152). which are taken from the same paper.

We apply an allowable onset-to-treatment time of 270 minutes for patients aged less than 80, and 180 minutes for patients aged 80+.

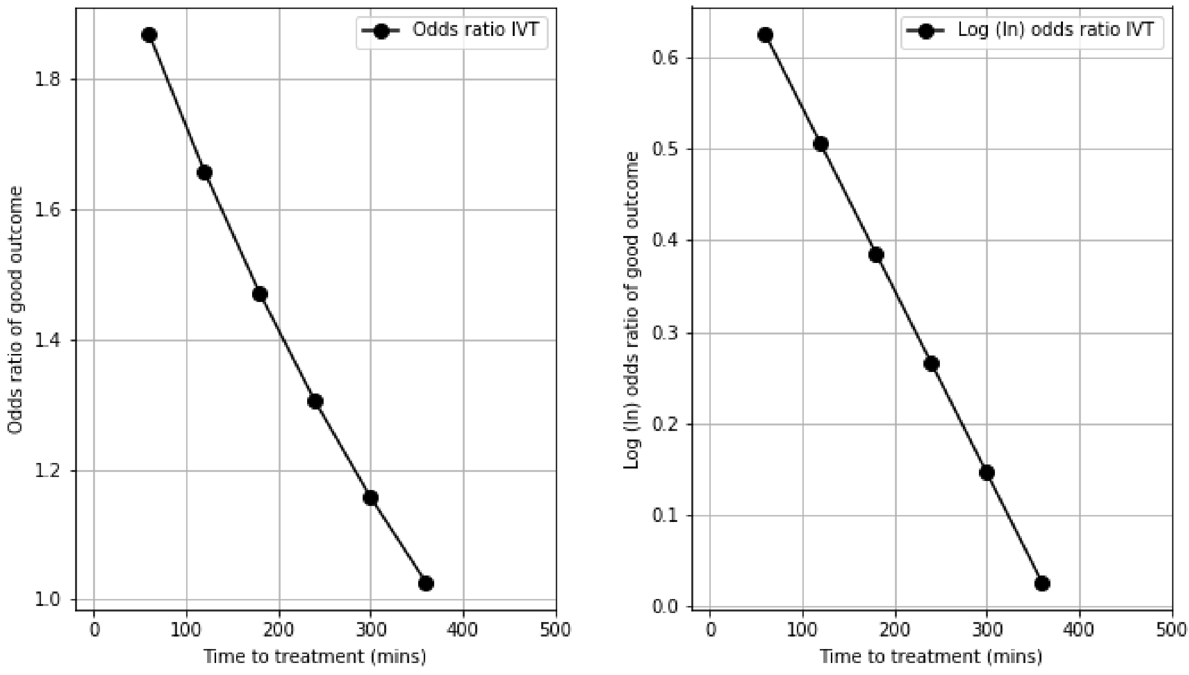


*Figure 2: The decay of effectiveness of IVT as a function of time to treatment, shown as decay in odds (left) or log odds (right).*

## 3.6 Model validation

The pathway simulation model was validated by 1) random bootstrap sampling varying overall thrombolysis use, comparing actual to predicted thrombolysis use, and 2) comparing actual and predicted thrombolysis use and speed across the seven hospitals.

In the random sampling (figure 3), 600 patients (typical of an acute stroke unit) were sampled randomly from either the thrombolysis group or the no-thrombolysis group, to give varying samples with an overall thrombolysis use rate of 1-25%. 100 model runs were performed. The model



For peer review only - http://bmjopen.bmj.com/site/about/guidelines.xhtml

showed very good correlation (R-square 0.99) between actual and predicted values though the model slightly under-predicted actual thrombolysis use with predicted thrombolysis being, on average, 89% that of actual thrombolysis use.

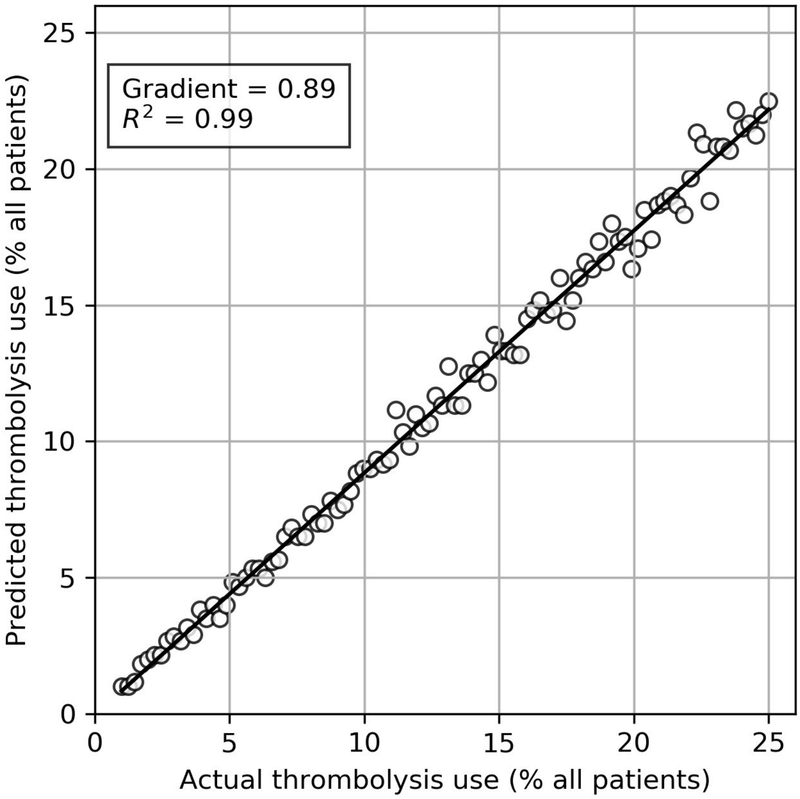


*Figure 3: Validation of the pathway simulation model, comparing actual to modelled (predicted) thrombolysis based on random sampling of all data. Samples were 600 points (representing typical sacute troke unit admission numbers) chosen randomly with resampling from patients given or not given thrombolysis to create a range of thrombolysis use examples. Points show mean predicted thrombolysis use for all confirmed stroke patients arriving at hospital, with bars showing 5th and 95th percentiles from 100 runs, with each run modelling one year.*

The model was further validated by comparing modelled (predicted) use of thrombolysis with actual use of thrombolysis. Actual use of thrombolysis was based on modelling a one year period, with replicates of 100 runs (each with different random number seeds) in order to determine expected year-to-year variation. The model showed very good correlation (R-square 0.96) between actual and predicted values though the model again slightly under-predicted actual thrombolysis use with predicted thrombolysis being, on average, 90% that of actual thrombolysis use (table 6).



For peer review only - http://bmjopen.bmj.com/site/about/guidelines.xhtml

*Table 6: Comparison of actual vs. modelled hospital performance*

| **Hospital** | **1** | **2** | **3** | **4** | **5** | **6** | **7** |
|---|---|---|---|---|---|---|---|
| Actual thrombolysis (%) | 13.7 | 8.0 | 8.4 | 7.0 | 8.5 | 14.5 | 9.2 |
| Model thrombolysis (%) | 12.9 | 7.1 | 7.6 | 6.3 | 8.1 | 12.0 | 8.0 |
| Actual onset to thrombolysis (hrs) | 2.4 | 2.6 | 2.9 | 2.5 | 2.3 | 2.5 | 2.5 |
| Model onset to thrombolysis (hrs) | 2.6 | 2.6 | 3.0 | 2.6 | 2.7 | 2.6 | 2.5 |

The difference between predicted and actual thrombolysis use is largely explained by the observation that 8% of thrombolysis in the SSNAP data set was given outside of the assumed allowable times for thrombolysis in the model (4.5 hrs for patients aged <80, and 3 hrs for patients aged 80+); the model applies a stricter time cut-off than clinicians allow in reality.

## 3.7 Alternative model scenarios

Though many different 'what if?' scenarios may be tested in the model we have reported on key alternative scenarios?

1. Door-to-needle time 30 minutes for 90% patients (the other 10% do not receive a scan within time to treat with thrombolysis). Door-to-needle time is split equally between arrival-to-scan and scan-to-thrombolysis?
2. Of those with ischaemic stroke, and who receive a scan with time to treat, 60% are treated with thrombolysis (from the analysis by Bembenek *et al*. [2]).
3. Stroke onset time is determined for 77% of patients (upper quartile point in SSNAP 2016/17).
4. Combinations of the above

Additionally, in place of scenario 2, thrombolysis rate may be determined according to the method based on machine learning (see below), to allow for stroke patient population differences between hospitals.

# 4 Machine learning

All machine learning methods and validation was coded in Python using the SciKit Learn machine learning [3].

All models are *supervised learning* models, and predict whether an individual patient would receive thrombolysis or not given a set of training data with features and known label (whether thrombolysis was given). The machine learning models evaluated are:

- Logistic regression with regularisation [4].
- Random Forests [5].



For peer review only - http://bmjopen.bmj.com/site/about/guidelines.xhtml

- Support Vector Machines: either a linear model was used, or a model using feature transformation by a radial basis function (rbf) kernel [6].
- A Feed-Forward Neural Network (results report a network based on two hidden layers of 50 and 5 nodes, after optimisation of the network size) [7].

Meta-parameters for each model may be found in the GitHub code repository, in the file machine_learning_all_techniques.py

## 4.1 Model features

Model features were set for each patient (anonymous data only was used) from SSNAP (Sentinel Stroke National Audit Programme).

The inclusion criteria for patients was the patient had been scanned with 30 minutes left to give thrombolysis (allowing 4.5 hours and 3 hours from onset to treatment for patients aged under 80, and 80+, respectively). Details of the individual fields are shown in table 7.

*Table 7: SSNAP fields used in machine learning model*

| Feature | Type | Feature | Type |
|---|---|---|---|
| Hosp_1 | Binary | Anticoag before stroke_1 | Binary |
| Hosp_2 | Binary | Anticoag before stroke_NK | Binary |
| Hosp_3 | Binary | Stroke severity group_1. No stroke symptoms | Binary |
| Hosp_4 | Binary | Stroke severity group_2. Minor | Binary |
| Hosp_5 | Binary | Stroke severity group_3. Moderate | Binary |
| Hosp_6 | Binary | Stroke severity group_4. Moderate to severe | Binary |
| Hosp_7 | Binary | Stroke severity group_5. Severe | Binary |
| Male | Binary | Stroke Type_I | Binary |
| Age (years) | Integer | Stroke Type_PIH | Binary |
| 80 | Binary | S2RankinBeforeStroke | Integer |
| Onset Time Known Type_BE | Binary | S2NihssArrival | Integer |
| Onset Time Known Type_NK | Binary | S2NihssArrivalLocQuestions | Integer |
| Onset Time Known Type_P | Binary | S2NihssArrivalLocCommands | Integer |
| # Comorbidities | Integer | S2NihssArrivalBestGaze | Integer |
| 2+ comorbidotes | Binary | S2NihssArrivalVisual | Integer |
| Congestive HF | Binary | S2NihssArrivalFacialPalsy | Integer |
| Hypertension | Binary | S2NihssArrivalMotorArmLeft | Integer |
| Atrial Fib | Binary | S2NihssArrivalMotorArmRight | Integer |
| Diabetes | Binary | S2NihssArrivalMotorLegLeft | Integer |
| TIA | Binary | S2NihssArrivalMotorLegRight | Integer |
| Co-mordity | Binary | S2NihssArrivalLimbAtaxia | Integer |
| Antiplatelet_0 | Binary | S2NihssArrivalSensory | Integer |
| Antiplatelet_1 | Binary | S2NihssArrivalBestLanguage | Integer |
| Antiplatelet_NK | Binary | S2NihssArrivalDysarthria | Integer |
| Anticoag before stroke_0 | Binary | S2NihssArrivalExtinctionInattention | Integer |

## 4.2 Machine learning validation

The machine learning model was validated using stratified ten-fold validation [8], where the data is split into 10 subsets, and the model run 10 times (with each model run using 9 subsets for training



For peer review only - http://bmjopen.bmj.com/site/about/guidelines.xhtml

and 1 subset held back for testing, with all data present in a test subset once and only once). For validation all hospitals were combined, with the hospital as an input parameter in the model.

Table 8 shows performance of different machine learning models (Support Vector Machines models were preformed with linear or radial basis function kernels). All models used the SciKit Learn library for Python. The Random Forest model was chosen for the work described in the paper.

The models were evaluated for:

- *Accuracy*: The proportion of thrombolysis decisions correctly predicted.
- *Sensitivity* (also called *recall* or *true positive rate)*: The proportion of positive thrombolysis decisions correctly predicted.
- *Specificity*: The proportion of negative thrombolysis decisions correctly predicted. The false *positive rate* is equal to 1-specificity.
- ROC area: The area of the operator-receiver characteristic curve, a plot of *true positive rate* against *false positive rate* [9].

*Table 8: Performance of machine learning models (Mean and standard deviation for 10 k-fold training/test splits). Random Forest was chosen for its highest overall accuracy and ROC.*

| | accuracy | sensitivity | specificity | ROC area |
|---|---|---|---|---|
| Logistic regression | 0.807 (0.010) | 0.788 (0.015) | 0.819 (0.012) | 0.882 (0.005) |
| **Random Forest** | **0.819 (0.010)** | **0.792 (0.017)** | **0.837 (0.011)** | **0.889 (0.007)** |
| SVM (linear) | 0.807 (0.008) | 0.820 (0.012) | 0.798 (0.012) | 0.880 (0.005) |
| SVM (rbf) | 0.813 (0.006) | 0.820 (0.013) | 0.808 (0.008) | 0.880 (0.006) |
| Neural Network | 0.807 (0.010) | 0.794 (0.017) | 0.816 (0.012) | 0.882 (0.005) |

Figure 4 shows the ROC for Random Forest model, which plots true positive rate (sensitivity, or recall) against false positive rate (1-specificity).



For peer review only - http://bmjopen.bmj.com/site/about/guidelines.xhtml

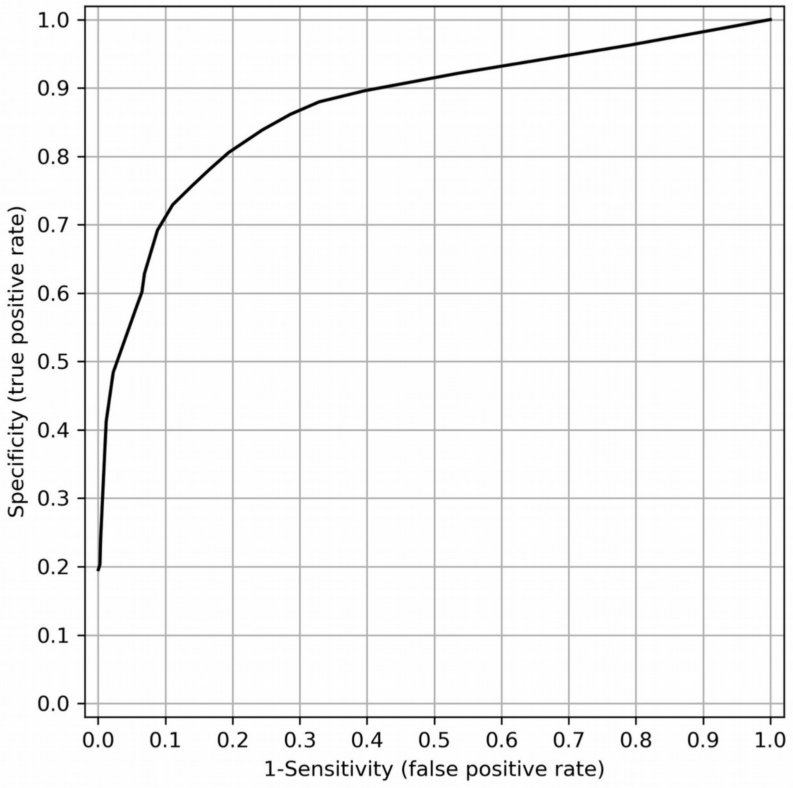


*Figure 4: Receiver Operator Curve (ROC) for the Random Forest model*

## 4.3 Learning curve for Random Forest

Figure 5 shows the learning curve for the Random Forest model. The learning rate shows how accuracy increases with the size of the training data set, and helps to establish whether more data is likely to lead to increased accuracy of the model. The accuracy of Random Forest model (assess by stratified 10-fold validation, with the training set reduced to the required size) increases rapidly to 79% accuracy with 400 data points, and then there is a shallow incline of improvement of accuracy up to our maximum available training data set size of 1600 data points. A larger data set may lead to further small increase in accuracy.



For peer review only - http://bmjopen.bmj.com/site/about/guidelines.xhtml

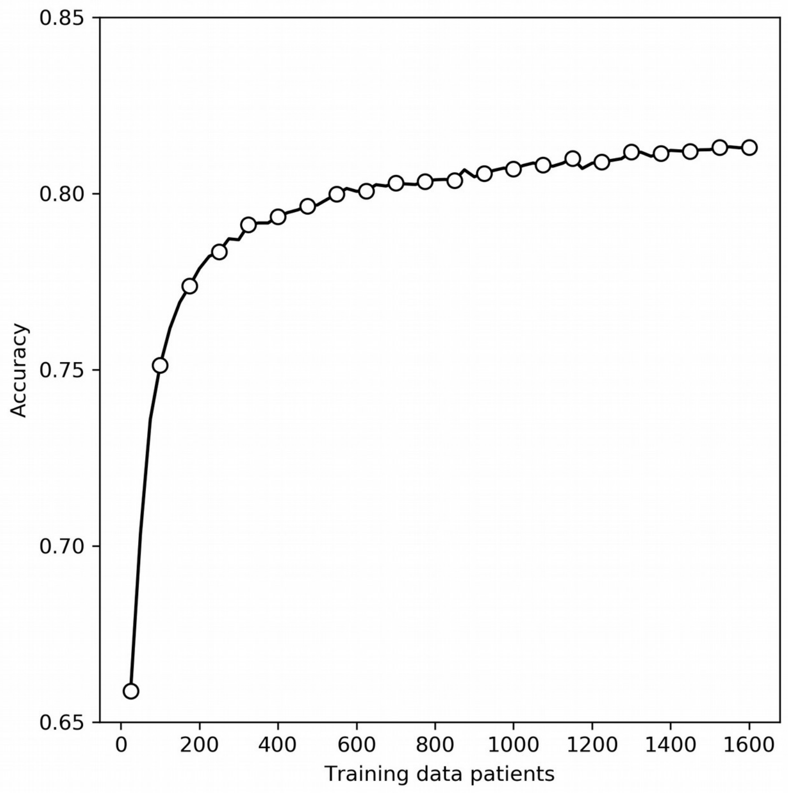


*Figure 5: Learning curve for the Random Forest model. Accuracy was measured using stratified 10-fold validation.*

## 4.4 Machine learning alternative scenarios

A machine learning model may be trained on a subset of patients to investigate how the difference in thrombolysis use between hospitals may be proportionally attributed to either the hospital or the local patient population. Table 9 shows the predicted use of thrombolysis in a set of patients that attend one hospital, based on decisions made from training at another hospital.

Taking hospital 7 as an example, the current thrombolysis use rate is 31% (for patients with 30 minutes left to receive thrombolysis). But of the Random Forest model is trained on decisions made at hospital 1, then 45% of the patients attending hospital 7 are predicted to receive thrombolysis. This is still lower than the 52% thrombolysis use rate in hospital 7, suggesting that the difference in thrombolysis rates between hospital 7 and 1 are due to differences in both decision-making (with hospital 1 being more inclined to use thrombolysis) and patients (with patients attending hospital 7 being less suitable for thrombolysis).

Alternatively the table shows the likely thrombolysis use rate at hospital 7 given alternative patient populations. If hospital 7 received patients currently attending hospital 1 then the model predicts thrombolysis use would be 40%. This is higher than the 31% current use of thrombolysis, but lower than the 52% current performance at hospital 1, again demonstrating that the use of thrombolysis depends on both the hospital and the patient cohort, and that not all hospitals should be expected to



For peer review only - http://bmjopen.bmj.com/site/about/guidelines.xhtml

achieve the same thrombolysis use rate (even when the patient cohort is restricted to those with time to thrombolyse). One possible explanation for these results is that the average stroke severity for hospital 7 is lower than than that for hospital 1 (9.0 vs 11.8, table 2), and that all hospitals may be less inclined to treat more mild strokes with thrombolysis, but that hospital 1 is more inclined than hospital 7 to treat more mild strokes with thrombolysis.

*Table 9: Predicted thrombolysis use (for patients with patients scanned with time left to thrombolysis) if the decision to give thrombolysis is based on decisions made by a Random Forest model trained at different hospitals. The columns therefore represent the likely difference in thrombolysis use due to differences in decision making. Hospital 7 is highlighted as an example described in the text.*

| | | **Actual thrombolysis use by hospital** | | | | | | |
|---|---|---|---|---|---|---|---|---|
| | | **1** | **2** | **3** | **4** | **5** | **6** | **7** |
| | | 52 | 35 | 48 | 33 | 49 | 44 | 31 |
| | | **Hospital patients actually attended** | | | | | | |
| | | 1 | 2 | 3 | 4 | 5 | 6 | **7** |
| **Hospital used to train model** | **1** | 52 | 42 | 58 | 50 | 67 | 57 | 45 |
| | **2** | 48 | 35 | 55 | 36 | 46 | 37 | 29 |
| | **3** | 53 | 38 | 48 | 46 | 58 | 41 | 34 |
| | **4** | 40 | 28 | 48 | 33 | 52 | 29 | 26 |
| | **5** | 50 | 36 | 50 | 40 | 49 | 45 | 37 |
| | **6** | 49 | 32 | 55 | 44 | 59 | 44 | 39 |
| | **7** | 42 | 23 | 42 | 31 | 50 | 36 | 31 |

It should be noted that table 9 represents predictions of *counter-factual scenarios* that can never be validated.

# 5 References


1 Emberson J, Lees KR, Lyden P, *et al.* Effect of treatment delay, age, and stroke severity on the effects of intravenous thrombolysis with alteplase for acute ischaemic stroke: A meta-analysis of individual patient data from randomised trials. *The Lancet* 2014;**384**:1929–1935. doi:10.1016/S0140-6736(14)60584-5

2 Bembenek J, Kobayashi A, Sandercock P, *et al.* How many patients might receive thrombolytic therapy in the light of the ECASS-3 and IST-3 data? *Int J Stroke* 2010;**5**:430–1. doi:10.1111/j.1747-4949.2010.00479.x

3 Pedregosa F, Varoquaux G, Gramfort A, *et al.* Scikit-learn: Machine Learning in Python. *J Mach Learn Res* 2011;**12**:2825–30.

4 Yu H-F, Huang F-L, Lin C-J. Dual coordinate descent methods for logistic regression and maximum entropy models. *Mach Learn* 2011;**85**:41–75. doi:10.1007/s10994-010-5221-8




For peer review only - http://bmjopen.bmj.com/site/about/guidelines.xhtml

For peer review only

5 Breiman, L. Random Forests. *Mach Learn*;**45**:5–32.

6 Fan R-E, Chang K-W, Hsieh C-J, *et al.* LIBLINEAR: A Library for Large Linear Classification. *J Mach Learn* 2008;**9**:1871–4.

7 Rumelhart, Daid E., Hinton, Geoffrey, E, Williams, Ronald J. Learning internal representations by error-propagation. In: *Parallel Distributed Processing: Explorations in the Microstructure of Cognition. Volume 1*. MIT Press, Cambridge, MA 1986. 318–62.

8 Stone, M. Cross-validatory Choice and Assessment of Statistical Predictions. *J R Stat Soc Ser B-Methodol* 1974;**36**:111–47.

9 Fawcett T. An introduction to ROC analysis. *Pattern Recognit Lett* 2006;**27**:861–74. doi:10.1016/j.patrec.2005.10.010

For peer review only



For peer review only - http://bmjopen.bmj.com/site/about/guidelines.xhtml
